# Identification of MicroRNAs from *Eugenia uniflora* by High-Throughput Sequencing and Bioinformatics Analysis

**DOI:** 10.1371/journal.pone.0049811

**Published:** 2012-11-15

**Authors:** Frank Guzman, Mauricio P. Almerão, Ana P. Körbes, Guilherme Loss-Morais, Rogerio Margis

**Affiliations:** 1 PPGGBM, Departamento de Genética, Universidade Federal do Rio Grande do Sul - UFRGS, Porto Alegre, Rio Grande do Sul, Brazil; 2 PPGBCM, Centro de Biotecnologia, Universidade Federal do Rio Grande do Sul - UFRGS, Porto Alegre, Rio Grande do Sul, Brazil; 3 Departamento de Biofisica, Universidade Federal do Rio Grande do Sul - UFRGS, Porto Alegre, Rio Grande do Sul, Brazil; Iwate University, Japan

## Abstract

**Background:**

microRNAs or miRNAs are small non-coding regulatory RNAs that play important functions in the regulation of gene expression at the post-transcriptional level by targeting mRNAs for degradation or inhibiting protein translation. *Eugenia uniflora* is a plant native to tropical America with pharmacological and ecological importance, and there have been no previous studies concerning its gene expression and regulation. To date, no miRNAs have been reported in Myrtaceae species.

**Results:**

Small RNA and RNA-seq libraries were constructed to identify miRNAs and pre-miRNAs in *Eugenia uniflora*. Solexa technology was used to perform high throughput sequencing of the library, and the data obtained were analyzed using bioinformatics tools. From 14,489,131 small RNA clean reads, we obtained 1,852,722 mature miRNA sequences representing 45 conserved families that have been identified in other plant species. Further analysis using contigs assembled from RNA-seq allowed the prediction of secondary structures of 25 known and 17 novel pre-miRNAs. The expression of twenty-seven identified miRNAs was also validated using RT-PCR assays. Potential targets were predicted for the most abundant mature miRNAs in the identified pre-miRNAs based on sequence homology.

**Conclusions:**

This study is the first large scale identification of miRNAs and their potential targets from a species of the Myrtaceae family without genomic sequence resources. Our study provides more information about the evolutionary conservation of the regulatory network of miRNAs in plants and highlights species-specific miRNAs.

## Introduction

*Eugenia uniflora* is a tropical fruit tree native to South America. The shrubby tree produces edible cherry-like fruits, which are locally known as pitanga or the Brazilian cherry. This species belongs to the Myrtaceae family, which is characterized by the presence of tannins, flavonoids, monoterpenes and sesquiterpenes whose presence and concentration varies between specimens from different geographical locations [1]–[3]. Extracts from pitanga leaves contain interesting biological properties that have been reported in several studies, and pitanga juice is used in folk medicine as a diuretic, antirheumatic, antipyretic, antidiarrhetic and antidiabetic [4]–[9]. *E. uniflora* is also an important ecological model to study because it grows in areas of medium and large levels of rainfall and can also be found in different vegetation types and ecosystems [10]. The variation in the metabolite concentration and the adaptability to different environments observed in *E. uniflora* indicating that these are the result of the transcriptional regulation of many genes involved in metabolic and signaling pathways.

MicroRNAs (miRNAs) are small non-coding regulatory RNAs widely found in unicellular and multicellular organisms that act as regulators of gene expression at the post-transcriptional level on genes containing miRNA target sites [11]. Mature miRNAs are single-stranded RNA molecules of approximately 21 nucleotides (nt) in length processed from a precursor molecule (pre-miRNA) [12]. To regulate protein-coding genes, the mature miRNA binds with perfect or imperfect complementarity to sites in the 5′ or 3′ untranslated regions (UTR) or coding sequences (CDS) of genes, which leads to mRNA degradation or translation inhibition [13]–[14]. In plants, miRNAs have diverse biological functions and are involved in the regulation of optimal growth and development as well as other physiological processes, including abiotic and biotic stress responses [15]. Several studies showed that many miRNAs are conserved across different plant families [16]–[17]. However, family- and species-specific miRNAs that are expressed in lower levels and probably have evolved more recently have been reported [18].

In the present study, in order to evaluate the importance of miRNAs in the regulation of gene expression and metabolic pathways in *E. uniflora,* we constructed small RNA (sRNA) and polyA RNA-seq libraries from leaves and sequenced the libraries with high throughput Solexa technology. The sequencing data were analyzed to identify conserved and novel miRNAs and their respective targets. This work represents the first report of miRNAs identified in Myrtaceae.

## Methods

### Plant Material and RNA Isolation

Total RNA was isolated from *E. uniflora* leaves using the CTAB method [19]. RNA quality was evaluated by electrophoresis on a 1% agarose gel, and quantification was determined using a NanoDrop spectrophotometer (NanoDrop Technologies, Wilmington, DE, USA).

### Deep Sequencing

Total RNA (>10 µg) was sent to Fasteris SA (Plan-les-Ouates, Switzerland) for processing. One sRNA library was constructed and sequenced using the Illumina HiSeq2000 platform. Briefly, the construction of the sRNA library consisted of the following successive steps: acrylamide gel purification of the RNA bands corresponding to a size range of 20–30 nt; ligation of the 3p and 5p adapters to the RNA in two separate subsequent steps, each followed by acrylamide gel purification; cDNA synthesis followed by acrylamide gel purification; and a final step of PCR amplification to generate a cDNA colony template library for Illumina sequencing.

The polyadenylated transcript sequencing (RNA-seq) was performed using the following successive steps: poly-A purification; cDNA synthesis using a poly-T primer shotgun method to generate inserts of 500 nt, 3p and 5p adapter ligations; pre-amplification; colony generation; and sequencing. The Illumina output data included sequence tags of 100 bases.

### Accession Numbers

Sequencing data are available at the NCBI Gene Expression Omnibus (GEO) ([http://www.ncbi.nlm.nih.gov/geo]). The accession number GSE38212 contains the sequence data from the RNA-seq and sRNA libraries derived from *E. uniflora* leaves.

### Data Analysis

The overall procedure for analyzing Illumina small libraries is shown in Figure S1. All low quality reads with FASTq values below 13 were removed, and 5′ and 3′ adapter sequences were trimmed using the Genome Analyzer Pipeline (Illumina) at Fasteris SA. The remaining low quality reads with ‘n’ were removed with PrinSeq script [20]. Sequences shorter than 18 nt and larger than 25 nt were excluded from further analysis. Small RNAs derived from Viridiplantae rRNAs, tRNAs, snRNAs and snoRNAs deposited at the tRNAdb [21], SILVA rRNA [22], and NONCODE v3.0 [23] databases and from Rosales mtDNA and cpDNA sequences deposited at the NCBI GenBank database [(http://ftp.ncbi.nlm.nih.gov)] were identified by mapping with Bowtie [24].

After cleaning the data (low quality reads, adapter sequences), the RNA-seq data were assembled into contigs using the CLC Genome Workbench version 4.0.2 (CLCbio, Aarhus, Denmark) algorithm for *de novo* sequence assembly using the default parameters (similarity = 0.8, length fraction = 0.5, insertion/deletion cost = 3, mismatch cost = 3). In total, 170,568 contigs were assembled and used as a reference for the discovery of pre-miRNA and target sequences.

### Identification of Conserved and Novel miRNAs

In order to determine conserved plant miRNAs, small RNA sequences were aligned with known non-redundant Magnoliophyta miRNAs deposited at miRBase (Release 18, November 2011) using Bowtie. Complete alignment of the sequences was required, and no mismatches were allowed. To search for novel miRNAs, small RNA sequences were matched against contigs obtained through *de novo* assembly of transcripts from mRNA sequences of *E. uniflora* leaves using SOAP2 [25]. The SOAP2 output was filtered with an in-house filter tool (FilterPrecursor) to identify candidate sequences as miRNA precursors using an anchoring pattern of one or two blocks of aligned small RNAs with perfect matches. The selected candidate precursors were manually inspected using the software Tablet [26] to visualize the presence of the anchoring pattern. As miRNA precursors have a characteristic hairpin structure, the next step to select candidate sequences included secondary structure analysis by RNAfold with the annotation algorithm from the UEA sRNA toolkit [27]. In addition, perfect stem-loop structures should have the miRNA sequence at one arm of the stem and a respective anti-sense sequence at the opposite arm. In the present study miRNAs were named in two different ways: (i) miR000, when corresponding to a family with two or more miRNA loci and (ii) MIR000, to design a single locus. Finally, precursor candidate sequences were checked using the BLAST algorithm from miRBase (www.mirbase.org).

### Validation of miRNA by RT-PCR

In order to validate predicted miRNAs, a series of RT-PCR were performed in RNA isolated from leaves of three individuals of *E. uniflora* occurring in the Grumari native protected area in Rio de Janeiro, Brazil. Among the analyzed miRNAs seventeen corresponded to conserved miRNAs (eun-MIR156, eun-MIR159, eun-MIR160, eun-MIR166, eun-MIR167-1, eun-MIR167-2, eun-MIR167-3, eun-MIR167-4, eun-MIR395, eun-MIR396-1, eun-MIR396-2, eun-MIR397-1, eun-MIR397-2, eun-MIR482-1, eun-MIR482-2, eun-MIR530, eun-MIR827) and ten were novel miRNAs (eun-MIR001-1, eun-MIR001-2, eun-MIR004-2, eun-MIR005, eun-MIR006, eun-MIR008, eun-MIR009, eun-MIR012, eun-MIR013, eun-MIR014). The stem-loop primer, used for miRNA cDNA synthesis, was designed according to Cheng *et al*. [28]. The forward miRNAs primers were designed based on the full mature miRNA sequences and the reverse primer was the universal reverse primer for miRNA. The RT-PCR was performed according the conditions used by Kulcheski *et al*. [29]. Briefly, reactions were completed in a volume of 20 µL containing 10 µL of diluted cDNA (1∶100), 0.025 mM dNTP, 1X PCR Buffer, 3 mM MgCl2, 0.25 U Hot Start Taq DNA Polymerase (Promega) and 200 nM of each reverse and forward primer. Samples were analyzed in biological triplicate in a 96-well plate, and a no-template control was included. The PCR conditions were performed in an ABI 7500 Real-Time PCR System (Applied Biosystems). The PCR products were resolved on a 2% agarose gel and analyzed using Quantity One software (Bio-Rad).

### Prediction of miRNA Targets

Previously assembled mRNA contigs were clustered using the Gene Indices Clustering Tools (http://compbio.dfci.harvard.edu/tgi/software/) [30] to reduce any sequence redundancy. The clustering output was passed to the CAP3 assembler [31] for multiple alignment and consensus building. Contigs that did not reach the set threshold and fell into any assembly remained as a list of singletons.

The prediction of target genes for the most abundant mature miRNAs from the conserved and novel pre-miRNAs was performed by psRNAtarget [32]. The program uses a 0–5 scale to indicate the complementarity between miRNA and their target, with the smaller numbers representing higher complementary and zero corresponding to a perfect complementation. Default parameters with an expectation value of 4 and *E. uniflora* assembled unigenes longer than 600 bp were used. Candidate RNA sequences were then annotated by assignment of putative gene descriptions based on sequence similarity with previously identified genes annotated with details deposited in the protein database of NR and the Swiss-Prot/Uniprot protein database using BLASTx implemented in blast2GO v2.3.5 software [33]. The annotation was improved by the analysis of conserved domains/families using the InterProScan tool and Gene Ontology terms as determined by the GOslim tool from blast2GO software. At the same time, the orientations of the transcripts were obtained from BLAST annotations.

Finally, to verify if the genes targeted by the identified miRNAs regulate any metabolic pathways involved in the secondary metabolites synthesis, we obtained the enzyme EC numbers for each target gene from the blast2GO annotation. These codes were uploaded to iPATH2 server [34] to generate metabolic pathway maps.

## Results

### *E. uniflora* RNA Library Sequencing

**Figure 1:**
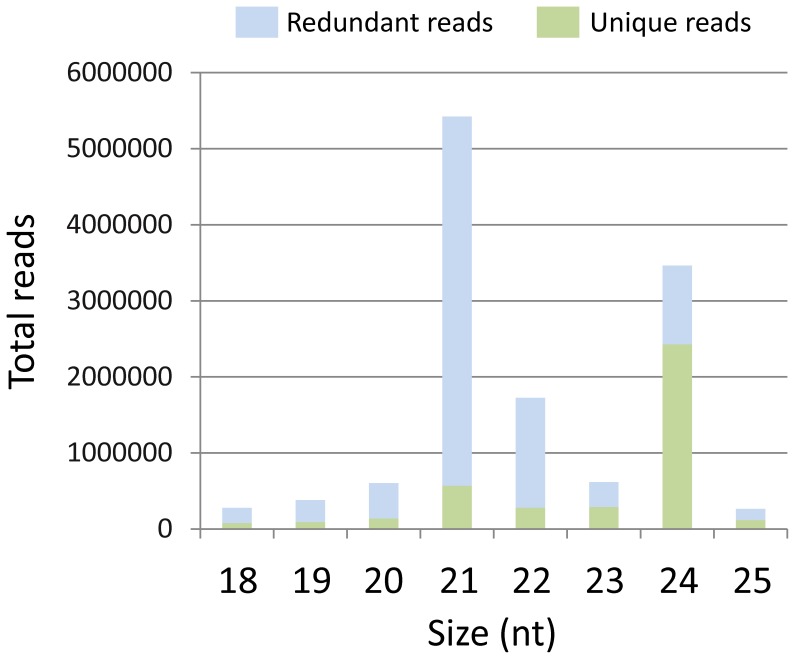
Length distribution and diversity of small RNA reads in the *E. uniflora* leaf library.

To identify conserved and novel miRNAs in *E. uniflora*, sRNA library was constructed from leaves and sequenced using Solexa high-throughput technology. After removing low quality sequences, those without inserts, or those with adapter contaminants or lengths outside of the 18–25 nt range, a total of 14,849,131 reads were obtained (Table 1). The number of reads with different lengths in the redundant and non-redundant sRNA datasets is shown in Figure 1 and Table S1. The most abundant sRNA species contained 21 nt, whereas the highest sequence diversity was observed in the 24-nt fraction. Approximately 6.55% of the reads matched other types of non-coding sRNAs, such as rRNAs, tRNAs, snRNAs or snoRNAs, and 9.45% matched organellar DNA (Table 2).

**Table 1:** Summary of data from sequencing of *E. uniflora* small RNA library.

Type	Number of reads	Percentage (%)
Total reads*	14,849,131	100
18–25 nt	12,759,506	86
<18 nt	1,554,975	10
>25 nt	534,650	4

* Reads with high quality with lenghts of 1 to 44 nt.

As there is no genome sequence available for *E. uniflora*, we sequenced the mRNA transcriptome of the *E. uniflora* leaf for use as a reference sequence in further analyses. The pooled mRNA-seq yielded 16,759,528 reads, which were imported into the CLC Genomics Workbench and *de novo* assembled into 170,568 contigs with an average length of 306 bp. Contigs and non-assembled reads with minimum lengths of 100 bp were further considered. The contigs ranged in size between the minimum set threshold of 100 bp and 7,808 bp (N50 = 447 bp), with 22,308 contigs more than 500 bp in length.

**Table 2:** Categorization of *E. uniflora* noncoding and organellar small RNAs*.

Small RNA type	Number of reads	Percentage (%)
miRNA	1,852,722	14.52%
rRNA	765,989	6.00%
tRNA	67,491	0.53%
snRNA	1,555	0.01%
snoRNA	859	0.01%
mtRNA	159,106	1.25%
cpRNA	1,046,305	8.20%
Other sRNA	8,865,479	69.48%

* 18–25 nt reads considered.

### Identification of Conserved miRNAs in *E. uniflora*

**Figure 2:**
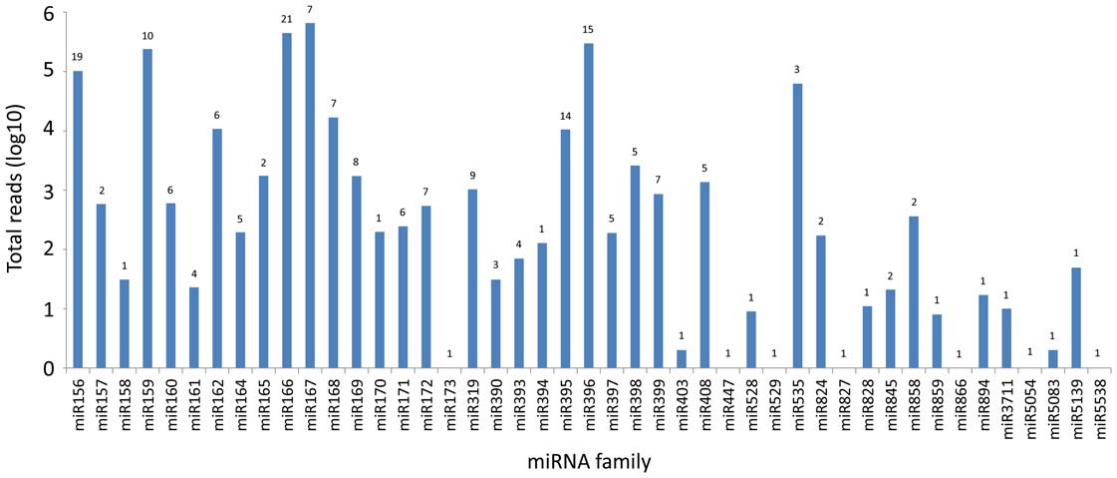
Number of identified miRNAs in each conserved miRNA family in plants. The values above the bars indicate the number of members identified in each conserved miRNA family.

There are 4,677 miRNAs from 47 Magnoliophyta species deposited in miRBase. To identify conserved miRNAs in *E. uniflora*, the small RNA library was matched against a set of 2,585 unique, mature plant miRNA sequences from the database. In total, 1,852,722 reads perfectly matched 204 known miRNAs (Figure 2 and Table S2). All identified sequences are distributed in 45 miRNA families, with an average of approximately 4 miRNA members per family. The largest family was miR166 with 21 members, which include isoforms found in several plant species. The miR156 (19 members), miR396 (15 members) and miR395 (14 members) families were the second, third and fourth largest miRNA families, respectively. Of the remaining miRNA families, 23 contained 2 to 10 members, and 18 were represented by a single member (Figure 2).

With respect to the abundance of each miRNA family, the frequencies varied from 1 read (7 families) to 656,093 reads (miR167), indicating that expression varies significantly among different miRNA families. This relative abundance is also observed in certain members from the same family. For example, the abundance of miR167 varied from 98 to 616,862 reads, as was the case for some other families, such as miR166 (1 to 381,733 reads), miR159 (2 to 235,279 reads) and miR396 (2 to 217,485 reads). These results indicate that different members have variable expression levels within one miRNA family.

Since the genome of *E. uniflora* is not publically available, the small RNA library was matched against a set of *de novo* assembled contigs from the *E. uniflora* leaf RNA-seq to identify putative miRNA precursor sequences. Candidate sequences with hairpin-like structures and mature miRNAs anchored in either or both of the 5p or 3p arms were further considered (Figure S2). Initial analysis allowed for the identification of 25 precursor sequences grouped into 15 conserved families (Table 3). The average value of MFE was -66.51 in these precursors and included two precursors (MIR167-2 and MIR169) with extreme values due to their long sizes. With respect to the % GC and MFEI, the average values were 47.63 and −0.94, respectively.

**Table 3:** Pre-miRNAs identified in *E. uniflora* with sequence similarities to plant conserved miRNA families.

miRNA	Precursor miRNA						Mature miRNA				
	Contig code	Length	GC %	MFE	AMFE	MFEI	5p sequence more abundant	Read count	3p more abundant sequence	Read count	Total reads
eun-MIR156	Contig92889	97	54.64	−55.20	−56.91	−1.04	TTGACAGAAGATAGAGAGCAC	83933	GCTCTCCCTCTCCTGTCAACA	1	85396
eun-MIR159	Contig93245	163	45.40	−55.86	−34.27	−0.75	AGCTGCTGGTCTATGGATCCC	376	CTTGCATATGCCAGGAGCTTC	493	1302
eun-MIR160	Contig81816	116	53.45	−54.30	−46.81	−0.88	TGCCTGGCTCCCTGTATGCCA	293	GCGTATGAGGAGCCAAGCATA	22	323
eun-MIR162	Contig165400	108	48.15	−35.80	−33.15	−0.69	GGAGGCAGCGGTTCATCGATC	24	TCGATAAACCTCTGCATCCAG	10713	10884
eun-MIR166	Contig94223	228	43.42	−72.40	−31.75	−0.73	GGAATGTTGTCTGGCTCGAGG	11016	TCGGACCAGGCTTCATTCCCC	381733	409546
eun-MIR167-1	Contig126350	90	45.56	−50.00	−55.56	−1.22	TGAAGCTGCCAGCATGATCTGA	616862	AGATCATCTGGCAGTTTCAAC	262	620306
eun-MIR167-2	Contig163487	615	37.40	−163.60	−26.60	−0.71	TGAAGCTGCCAGCATGATCTGG	32188	TCAGGTCATCTTGCAGCTTCA	939	34461
eun-MIR167-3	Contig784s	81	48.15	−38.60	−47.65	−0.99	TGAAGCTGCCAGCGTGATCTCA	16305	ATCAGATCATGTGGCAGCTTCACC	73	22056
eun-MIR1674	Contig784a	81	48.15	−38.70	−47.78	−0.99	TGAAGCTGCCACATGATCTGA	71	ND	−	72
eun-MIR169	Contig142088	730	39.18	−160.80	−22.03	−0.56	TTATAGGCGATTGGAGGTATG	876	TTAGCTAAAGTCGTCTTGCCCA	6818	8961
eun-MIR172-1	Contig83802	122	47.54	−55.10	−45.16	−0.95	CAGGTGTAGCATCATCAAGAT	36	AGAATCTTGATGATGCTGCAT	495	1076
eun-MIR172-2	Contig113567	92	42.39	−39.70	−43.15	−1.02	GCAGCATCATCAAGATTCACA	12	AGAATCTTGATGATGCTGCAT	495	523
eun-MIR172-3	Contig85928	165	43.64	−71.20	−43.15	−0.99	GTAGCATCATCAAGATTCACA	33	AGAATCTTGATGATGCTGCAT	495	1048
eun-MIR395	Contig114717	88	51.14	−47.50	−53.98	−1.06	TCCCCTAGAGTTCTCCTGAACA	107	ATGAAGTGTTTGGGGGAACTC	1356	1659
eun-MIR396-1	Contig94388	160	42.50	−68.60	−42.88	−1.01	TTCCACGGCTTTCTTGAACTG	217485	GTTCAATAAAGCTGTGGGAAG	2028	223828
eun-MIR396-2	Contig153308	128	42.19	−49.10	−38.36	−0.91	TTCCACAGCTTTCTTGAACTG	23061	GTTCAAGCTAGCTGTGGGAAG	12981	64439
eun-MIR397-1	Contig87345s	126	51.59	−68.80	−54.60	−1.06	TCATTGAGTGCAGCGTTGAT	626	CGGTTTCGACAGCGCTGCACT	59	1052
eun-MIR397-2	Contig87345a	126	51.59	−63.20	−50.16	−0.97	TGCAGCGCTGTCGAAACCGAT	20	TCAACGCTGCACTCAATGATG	273	322
eun-MIR482-1	Contig88445	153	53.90	−94.60	−61.43	−1.14	CATGGGTTGTTTGGTGAGAGG	24202	TCTTGCCAATACCACCCATGCC	70833	100235
eun-MIR482-2	Contig88445	139	53.96	−71.00	−51.08	−0.95	GAAATGGGAGGGTGGGAAAGA	982	TTTCCTATTCCTCCCATTCCAT	3371	5574
eun-MIR482-3	Contig85065	169	50.89	−92.40	−54.67	−1.07	GAGATTCGAGCTACCGGAAGTTGTG	329	TTCCCAAGGCCGCCCATTCCGA	14915	17039
eun-MIR530	Contig18750	183	51.37	−82.30	−44.97	−0.88	TCTGCATTTGCACCTGCACCT	185	AGGTGCGGGTGCAGGTGCAGA	12	280
eun-MIR535-1	Contig68094	102	50.00	−42.60	−41.76	−0.84	TGACAACGAGAGAGAGCACGC	62562	GTGCTCTCTATCGCTGTCATA	4199	76334
eun-MIR535-2	Contig71803	100	49.00	−47.00	−47.00	−0.96	TGACAACGAGAGAGAGCACGC	62562	TGCTCTCTACCGTTGTCATG	116	72263
eun-MIR827	Contig93928	81	45.68	−44.30	−54.69	−1.20	CTTTGTTGATGGCCATCTAATC	27	TTAGATGACCATCAGCGAACA	266	304

MFE: minimal folding free energy (kcal/mol); AMFE: Adjusted MFE; MFEI: minimal folding free energy index; ND: no detected.

**Figure 3:**
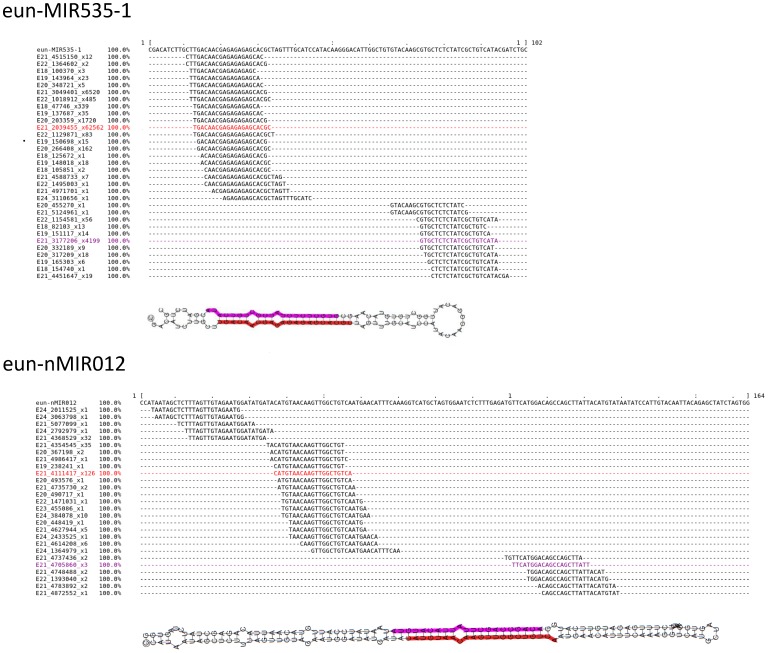
Predicted secondary structures of conserved and novel miRNAs of *E. uniflora*. Secondary structures of the precursors eun-MIR535-1 and eun-nMIR012, their locations and the expression of small RNAs mapped onto these precursors. Sequences of the most abundant mature miRNAs in the 5p and 3p arms are labeled in purple and red, respectively. Values on the left side of the miRNA sequence represent read counts in the leaf library.

Within the identified families, MIR167 was the most abundant, with 676,895 reads, and contained 4 members (MIR167-1, MIR167-2, MIR167-3 and MIR167-4). In addition, several miRNA isoforms were detected in the libraries, and several of these were more abundant than the known miRNAs reported in miRBase for other plants (Figure 3). Furthermore, in the family MIR397, one precursor was identified with a typical structure and mature reads in the sense and antisense orientations. Both orientations were considered two independent precursors from the same family for the following analysis.

### Identification of Novel miRNAs in *E. uniflora*

Using the previously described criteria in the identification of conserved pre-miRNAs, we obtained another 17 potential miRNA candidates grouped into 15 families (Table 4). In addition to the hairpin structure, the detection of miRNA* in 14 precursors is a strong indication to consider these miRNAs as true candidates. Comparisons among the mature sequences of candidate miRNAs and those miRNAs deposited in miRBase suggest that these candidates are novel miRNAs that have not been identified in others species and are possibly specific to the Myrtaceae family. These novel miRNAs displayed an average negative folding value of −137.89, which included 4 miRNAs with long sizes similarly observed in some previously identified conserved pre-miRNAs. With respect to the % GC and MFEI, the average values were 42.86 and −1.05, respectively. In addition, one novel pre-miRNA was found with mature sequences in the sense and antisense orientations and was considered to represent 2 members of the same family (nMIR001-1 and −2).

**Table 4:** New putative miRNA precursors identified in *E. uniflora*.

miRNA	Precursor miRNA						Mature miRNA				
	Contig name	Length	GC %	MFE	AMFE	MFEI	5p more abundant sequence	Read count	3p more abundant sequence	Read count	Total reads
eun-nMIR001-1	Contig164780s	820	54.39	−451.70	−55.09	−1.01	TCGGCTGTCAATTTCTGGATT	185919	ATCCAGAAATTGGCAGCCGTT	110	192103
eun-nMIR001-2	Contig164780a	820	54.39	−446.00	−54.39	−1.00	CAATTTCTGGATTTCAGTTCG	20	TCGAACTGAAATCCAGAAATT	2	63
eun-nMIR002	Contig29785	173	30.06	−56.70	−32.77	−1.09	CGAAAAATGATTGGTTGTATCGCT	18	CGATCTAATCAATCATTTTTCGGG	2	21
eun-nMIR003	Contig121100	110	48.18	−63.20	−57.45	−1.19	TACTCGTTCCGTTGATCCATC	88	TGGATCAATAGAACGAGCAGGTGA	157	561
eun-nMIR004-1	Contig37387s	104	29.81	−47.10	−45.29	−1.52	TCGTAAATCCACTATATCTCT	3	TAGATATAGTGGATTTTCGAT	9	13
eun-nMIR004-2	Contig37387a	104	29.81	−46.40	−44.62	−1.50	ND	−	CAGAGATATAGTGGATTTACG	314	385
eun-nMIR005	Contig143563	106	34.91	−21.40	−20.19	−0.58	ND	−	GAGAATGATGAGTTAAATGGA	12	30
eun-nMIR006	Contig113617	113	33.63	−42.70	−37.79	−1.12	TCTCTGTTGATCTGATAAATA	19	TTTGTCGGATGAACAGGGAAT	18	55
eun-nMIR007	Contig116664r	96	46.88	−55.80	−58.13	−1.24	TAGGGTCAGATCGCTACTTAG	211	TAAGTGGTGATCTGACTCTAA	4446	5750
eun-nMIR008	Contig79716	426	43.66	−228.00	−53.52	−1.23	TCGAGCCCCTCCCACAGATTG	313	ATCTGTGGAAGAGACTCGACT	8403	13617
eun-nMIR009	Contig81320	1545	37.41	−397.90	−25.75	−0.69	TTCAAGTCTAACAACCTCAGCT	5774	TGGAGGTTGTTTGGCTTGAGCT	1968	11577
eun-nMIR010	Contig84248	350	48.86	−143.30	−40.94	−0.84	TGCTGTTCTTCCGTTCACGAAT	309	TCGTGAAGGAAGAATGTGCAAT	6340	10307
eun-nMIR011	Contig164559	207	51.21	−98.70	−47.68	−0.93	GCTCGAGGTCAGTTTGTCGCC	1553	CGGCAAACTGGACCTCGAGATC	110	2884
eun-nMIR012	Contig167957	164	35.98	−91.80	−55.98	−1.56	CATGTAACAAGTTGGCTGTCA	126	TTCATGGACAGCCAGCTTATT	3	243
eun-nMIR013	Contig87665	179	56.42	−84.90	−47.43	−0.84	TGAAGCAGATCAAGAACCCAG	155	TCTCGTTCCGCTTCATCTGAA	2	172
eun-nMIR014	Contig200629r	87	55.17	−23.40	−26.90	−0.49	ND	−	GCATCACTAGCTTACGCTCTG	30	159
eun-nMIR015	Contig165886	124	37.90	−45.10	−36.37	−0.96	ND	−	CAATGAACGCATTTGCAGGTG	21	22

MFE: minimal folding free energy (kcal/mol); AMFE: Adjusted MFE; MFEI: minimal folding free energy index; ND: no detected.

### Biological Confirmation of Identified miRNAs in *E. uniflora*

The stem-loop RT-PCR method was used to validate the expression of seventeen conserved miRNAs (eun-MIR156, eun-MIR159, eun-MIR160, eun-MIR166, eun-MIR167-1, eun-MIR167-2, eun-MIR167-3, eun-MIR167-4, eun-MIR395, eun-MIR396-1, eun-MIR396-2, eun-MIR397-1, eun-MIR397-2, eun-MIR482-1, eun-MIR482-2, eun-MIR530, eun-MIR827) and ten novel miRNAs (eun-MIR001-1, eun-MIR001-2, eun-MIR004-2, eun-MIR005, eun-MIR006, eun-MIR008, eun-MIR009, eun-MIR012, eun-MIR013, eun-MIR014). We confirmed that these miRNAs were expressed in three different individuals collected *in situ* (Figure S3).

### Identification and Classification of miRNA Targets

To understand the biological function of miRNAs in *E. uniflora*, the putative mRNA target sites of miRNA candidates were identified by aligning the most abundant mature miRNAs of each conserved and novel precursor to a set of *E. uniflora* assembled unigenes using psRNA target with default parameters and a maximum expectation value of 4. We found 87 potential targets in total, where 52 were targets of conserved miRNAs and 35 were targets of novel miRNAs, with an approximate average of 3 targets per miRNA. Detailed annotation results are given in Table 5 and S3.

**Table 5:** Predicted targets of novel miRNAs in *E. uniflora.*

miRNA	Inhibition	Score*	Putative Function
eun-nMIR001	Cleavage	1.5	Atp-dependent helicase rhp16-like
	Cleavage	3	Cytochrome p450
	Cleavage	3.5	Long chain acyl- synthetase 9
	Cleavage	3.5	Kinesin-related protein
	Translation	3	Transcription initiation factor iib
	Translation	3	Brassinazole-resistant 1
	Translation	3.5	Myosin family protein with dil domain
eun-nMIR002	Cleavage	3	Serine threonine-protein phosphatase 2a regulatory subunit b subunit alpha-like
	Cleavage	3.5	Probable receptor-like protein kinase at1g67000-like
eun-nMIR003	Cleavage	4	Udp-glycosyltransferase 74b1
eun-nMIR004	Cleavage	2.5	Auxin efflux carrier protein
	Cleavage	3.5	Sucrose nonfermenting 4-like
	Translation	3.5	Type i inositol- -trisphosphate 5-phosphatase 2-like
eun-nMIR005	Translation	3	Pentatricopeptide repeat-containing protein
	Cleavage	3.5	Protein reticulata-related 1
	Cleavage	3.5	Agenet domain-containing protein
eun-nMIR006	Translation	3.5	Outward rectifying potassium channel
eun-nMIR007	Cleavage	3.5	Aspartate semialdehyde
	Cleavage	3	Primary-amine oxidase
eun-nMIR008	Translation	3.5	Adenosine deaminase
eun-nMIR009	Cleavage	3	Cc-nbs-lrr resistance protein
eun-nMIR010	Translation	4	P8mtcp1
	Cleavage	4	Nbs-lrr resistance protein
	Translation	4	Cullin-1-like isoform 1
eun-nMIR011	Translation	3	E3 ubiquitin-protein ligase upl7
	Cleavage	3.5	Eukaryotic peptide chain release factor subunit 1-1
eun-nMIR012	Translation	3.5	Tho complex subunit 2
eun-nMIR013	Translation	3.5	Beta-amylase
	Translation	3.5	Integral membrane single c2 domain protein
eun-nMIR014	Cleavage	0	Ycf68 protein
eun-nMIR015	Cleavage	3.5	Rna-binding motif x-linked 2
	Cleavage	3.5	Transducin wd-40 repeat-containing protein
	Cleavage	3.5	Clip-associating protein
	Cleavage	3.5	Probable exocyst complex component 4-like
	Cleavage	3.5	Photosystem i p700 apoprotein a1

* psRNATarget value.

Among the most important miRNA targets, also previously identified in other plants, we found the squamosa promoter binding protein (SBP)-like (SPL) genes, which are targets of the miR156 family and have functions that are conserved across plant species [17], affecting diverse developmental processes, such as leaf development, shoot maturation, phase change and flowering in plants [35]-[40]. We also identified the auxin response factor (ARF), a plant-specific family of DNA binding proteins involved in hormone signal transduction that are targets for the miRNA families miR167 and miR160 [15], [41], [42]. Another important gene identified and targeted by miR162, with a significant role in the regulation of gene expression, is the pentatricopeptide repeat gene (PPR). This gene belongs to a large family implicated in post-transcriptional processes, such as splicing, editing, processing and translation specifically in organelles like mitochondria and chloroplasts [43]. These results substantiate the in silico identification of conserved and novel targets from *E. uniflora*.

**Figure 4:**
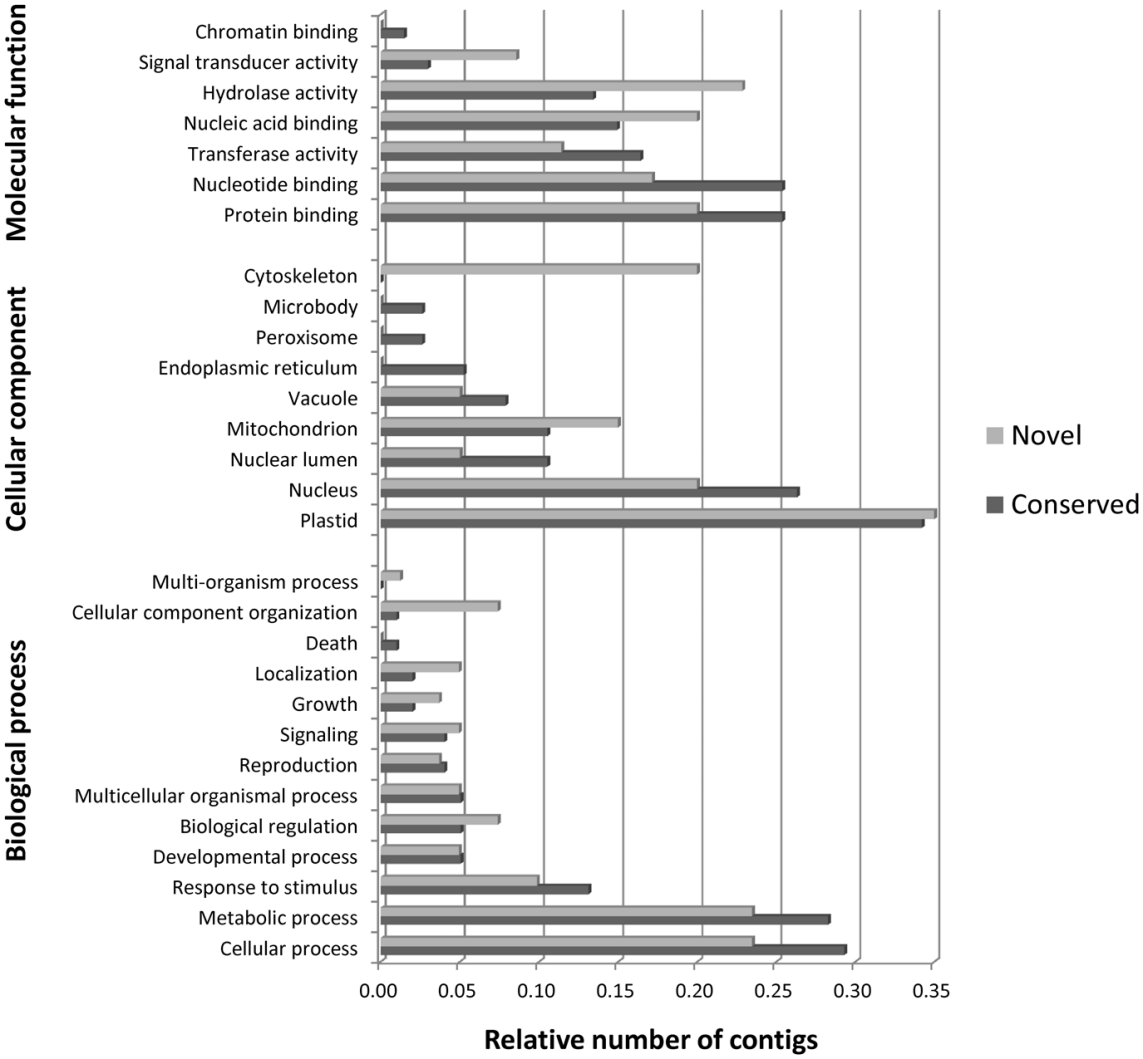
Gene categories and the distribution of target genes of the most abundant mature miRNAs in the conserved and novel pre-miRNA identified in *E. uniflora*.

All targets regulated by the conserved and novel miRNAs identified in this study were subjected to GO analysis to evaluate their potential functions. The categorization of these genes, according to biological processes, cellular components and molecular functions, is summarized in Figure 4. Based on biological processes, these targets were classified into 13 categories, and the three most overrepresented GO terms, either for conserved or novel miRNAs, were cellular processes, metabolic processes and responses to stimulus, suggesting that *Eugenia* miRNAs are involved in a broad range of physiological functions. Categories based on molecular function revealed that the target genes were related to 7 functions, and the four most frequent terms were protein binding, nucleotide binding, hydrolase activity and nucleic acid binding. In the category of cellular components, the analysis revealed that the protein products from the genes targeted by conserved and novel miRNAs are expressed mainly in the plastid and nucleus.

The iPATH2 server was used to produce an overview of the metabolic pathways involved in the secondary metabolites synthesis and potentially regulated by miRNAs in *E. uniflora*. Our results showed that three enzymes involved in several types of metabolism and secondary metabolites are regulated by identified miRNAs (Figure S4). The phosphoglycerate mutase is a potential target of eun-MIR396-2 and is involved in the pathway of gluconeogenesis while the hydroxyphenylpyruvate reductase is targeted by eun-MIR162 and is involved in terpenoid-quinone, tropane, pireridine and pyridine biosynthesis. In a similar way, eun-nMIR007 regulates primary-amine oxidase, an enzyme involved in the tropane, pireridine, pyridine and isoquinoline alkaloid biosynthesis.

## Discussion

Though several miRNAs have been identified via computational or experimental approaches in different plant families, there is no sequence or functional information available about miRNAs in any Myrtaceae species, which are economically important in the spice, fruit, timber and pharmacology industries [44].

We used Solexa technology for deep sequencing of a small RNA library to identify miRNAs in *E. uniflora*. The length distribution pattern obtained indicates that the majority of the redundant small RNAs from the library were 21 nt in length, which is atypical because 24 nt is the most abundant size produced by DCL3 in other plants [45]. This distribution pattern is similar to those observed in previous reports of plant small RNA sequencing using Solexa technology, such as wheat [46], grapevine [47], melon [48] and trifoliate orange [49], suggesting that the composition of the small RNA population varies among species. Additionally, other important causes for this variation include the developmental stage and environmental conditions in which the sample was collected. Contrary to the results observed with the redundant sequences, the analysis of the unique sequences showed that 24 nt was the dominant read length in comparison to all other sequence lengths, and similar results have been observed in other studies [50]-[53]. Small RNAs of 24 nt in length are known to be involved in heterochromatin transcriptional silencing in genomes with a high content of repetitive sequences [54], indicating the possible genome complexity of *E. uniflora*.

In this study, we compared our small RNA library from *E. uniflora* against known plant miRNAs from the miRBase database and identified 204 conserved miRNAs from different species grouped into 45 families. High throughput sequencing, which has the ability to generate millions of small RNA sequences, is a powerful tool to estimate expression profiles of miRNA. This technology provides the resources to determine the abundance of various miRNA families and even distinguish among different members of a given family. In our case, we found significant differences among the number and abundance of the members identified in each family, which is in agreement with previous studies [48], [55], [56] and suggests that this wide variation is due to a functional divergence in the conserved miRNA families.

Although conserved miRNAs have been identified by sequencing and comparison against miRNAs from other species, most plant species-specific miRNAs remain unidentified due to their lower levels of expression, which result in a small number of sequenced reads in comparison to the conserved miRNAs [57]. For this reason, we used a new approach to identify novel miRNAs in species where genomic data and resources were not available. We made use of simultaneous sequence comparison of small RNA and RNAseq libraries. Using this methodology, we identified 17 potential miRNA candidates specific for *E. uniflora*. From these, 14 contained complementary antisense miRNA, which provided more evidence for their existence as novel miRNAs, as observed in cucumber [51] and grapevine [53]. The other miRNAs that do not satisfy this last criterion require further investigation for their confirmation as miRNAs.

To understand the function of the identified miRNAs, their putative targets were predicted using a bioinformatics approach. Several identified targets of conserved miRNAs of *E. uniflora* are transcriptional factors, similar to the results reported in other studies [47], [48], [58], [59]. In the case of the novel miRNA targets, we found that the transcription initiation factor iib and the pentatricopeptide repeat-containing proteins are targeted by eun-nMIR001 and eun-nMIR005, respectively.

It has been reported in *Arabidopsis* that regulation of *ARF17* by miR160 is important for growth and development [60], regulation of *ARF6* and *8* by miR167 is important for development of anthers and ovules [61] and regulation of *ARF10* and *16* by miR160 plays a role in root cap formation [62]. In the present study, we found that *ARF17* is regulated by eun-MIR160 while other members of ARF family were not targeted by eun-MIR167. This discrepancy agrees with the previously reported in *Arabidopsis* because we used a leaf transcriptome as reference for the target identification. We confirm this observation not found homologs for *AtARF6*, 8, *10* and *16* by BLASTx in *E. uniflora* transcriptome.

In addition, with the analysis of GO terms, we identified 3 candidate targets likely involved in the response to abiotic stress: ATP-dependent helicase rhp16-like (eun-nMIR002), sucrose nonfermenting 4-like (eun-nMIR004) and serine threonine-protein phosphatase 2a regulatory subunit b'' subunit alpha-like (eun-nMIR002). The sucrose nonfermenting 4-like (SNF4) protein is a subunit of the probable trimeric SNF1-related protein kinase (SnRK) complex, which may play a role in a signal transduction cascade regulating gene expression and carbohydrate metabolism in higher plants [63]. Otherwise, the serine threonine-protein phosphatase 2A regulatory subunit b ''subunit alpha-like PP2Ab′′ is a structural subunit of the Ser/Thr phosphatases holoenzyme (PP2A) and recent studies suggesting the possible physiological role of PP2A in the drought stress response [64]. These results indicated that the targets from novel miRNAs identified here are possibly related to the adaptation of *E. uniflora* to different types of stress and environmental conditions observed *in natura*. Future experimental validation will determine how many of these predicted targets are genuinely targeted by miRNAs in specific environmental and physiological conditions.

Interestingly, we found three miRNAs involved in the regulation of enzymes that play critical roles in secondary metabolites synthesis. These findings suggest that variation in the levels of expression of these miRNAs could alter the levels of production of certain types of secondary metabolites. It is consistent with the previous reports that the concentration of these metabolites varies between specimens of *E. uniflora* from different geographical locations [2], [3]. More studies are necessary to confirm these preliminary findings and evaluate the correlation between the miRNA expression and secondary metabolite production.

### Conclusions

In summary, this study provides the first view of the diversity of miRNAs and their abundance in Myrtaceae and strongly supports the idea that miRNAs play an important conserved role in several physiological processes, as previously proposed for other plants. Our bioinformatics analysis indicates that miRNAs might contribute to different processes by affecting multiple target genes and different signaling pathways. Although the exact function of these miRNA target genes remains to be confirmed, we believe the present study provides novel insights into the molecular processes involved in conserved miRNA function.

## Supporting Information

Figure S1 **Flow chart of procedures for miRNA identification.**
(TIF)

Figure S2 **Represetation of the predicted secondary structures of the conserved and novel miRNA precursors of** ***E. uniflora*** **and the locations of the more abundant mature miRNAs.**
(PDF)

Figure S3 **Detection of miRNA expression in different** ***E. uniflora*** **individuals by RT-PCR.** Products generated by stem-loop RT-PCR were resolved on a 2% agarose. Leaf samples from three independent Eugenia uniflora trees were used to evaluate the presence of each miRNA.
(TIFF)

Figure S4 **iPath secondary metabolite map showing the different pathways where are involved each evaluated enzyme.** Each grey dot represents a metabolite and each colored line represents the different route affected by the enzyme targeted. In red: phosphoglycerate mutase (regulated by eun-MIR396-2). In blue: primary-amine oxidase (regulated by eun-nMIR007).
(TIFF)

Table S1 **Length distribution and read sequence abundance in the** ***E. uniflora*** **small RNA library.**
(XLS)

Table S2 **Abundance of predicted plant conserved miRNAs in the small RNA library of** ***E. uniflora*** **.**
(XLS)

Table S3 **Predicted transcript targets of plant conserved miRNAs in** ***E. uniflora*** **.**
(XLS)
