# Supplementary figures and images for: Identification of MicroRNAs from Eugenia uniflora by High-Throughput Sequencing and Bioinformatics Analysis
Source: PLoS One. 2012 Nov 15;7(11):e49811. doi: 10.1371/journal.pone.0049811 (PMC3499529; doi:10.1371/journal.pone.0049811)

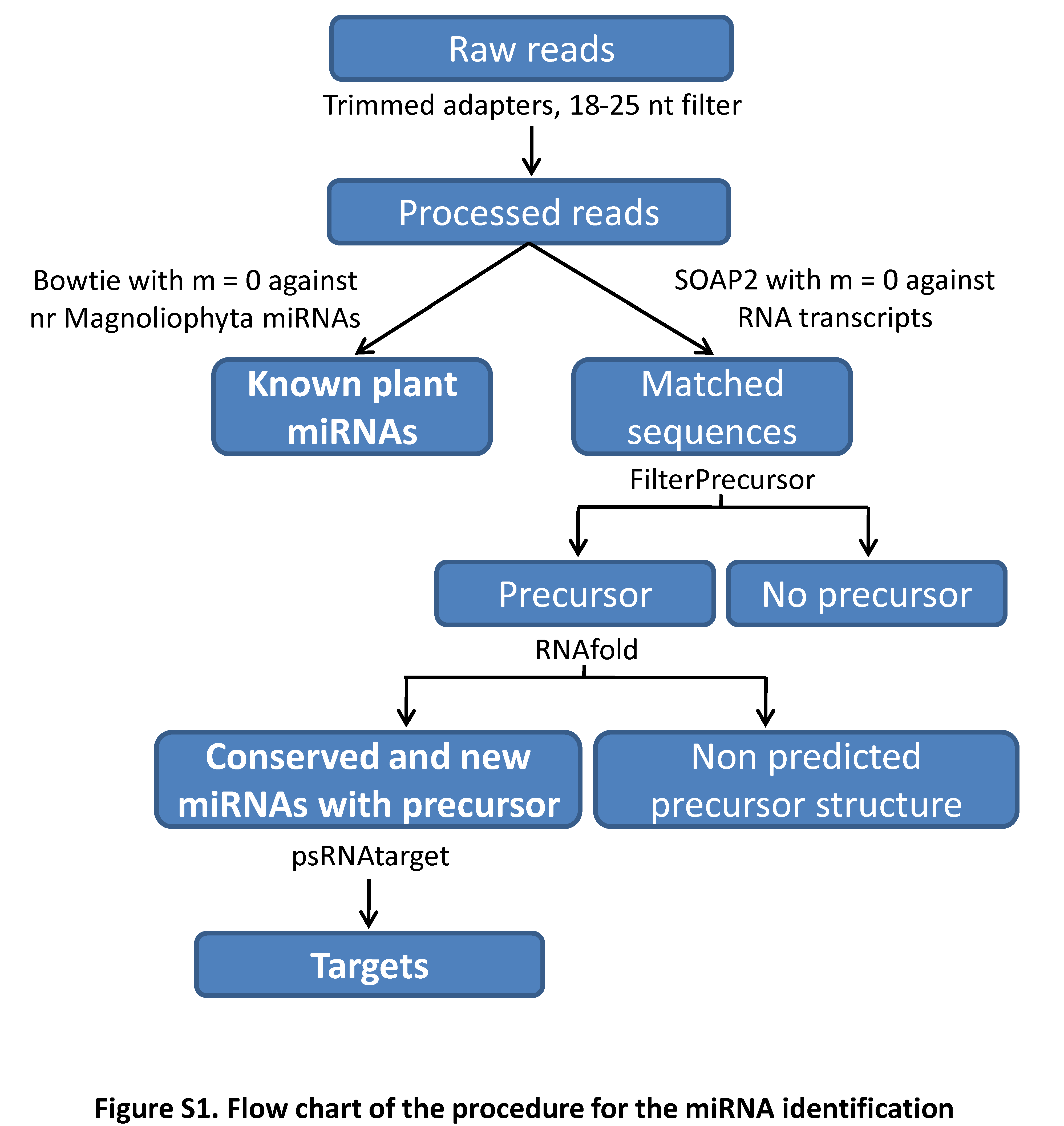

Supplement: Figure S1 — Flow chart of procedures for miRNA identification. (TIF) [file pone.0049811.s001.tif]

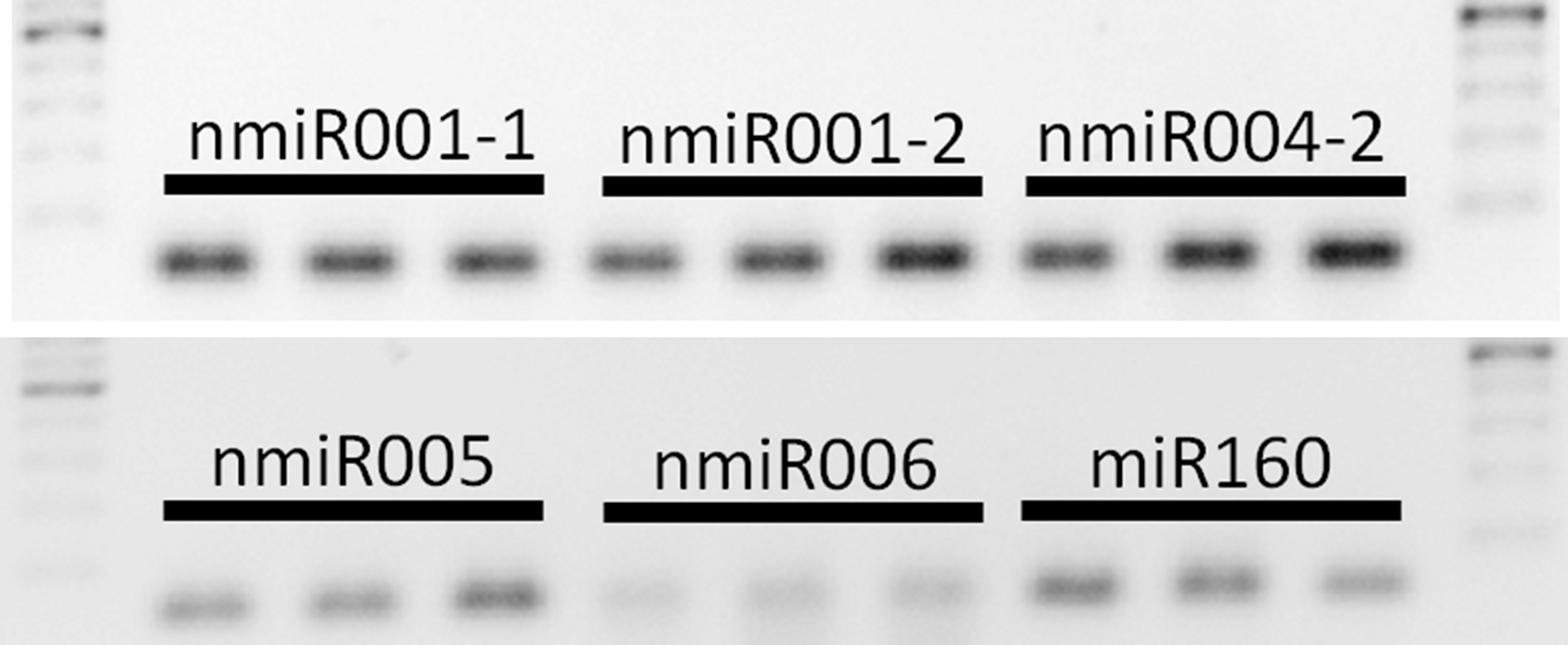

Supplement: Figure S3 — Detection of miRNA expression in different E. uniflora individuals by RT-PCR. Products generated by stem-loop RT-PCR were resolved on a 2% agarose. Leaf samples from three independent Eugenia uniflora trees were used to evaluate the presence of each miRNA. (TIFF) [file pone.0049811.s003.tiff]

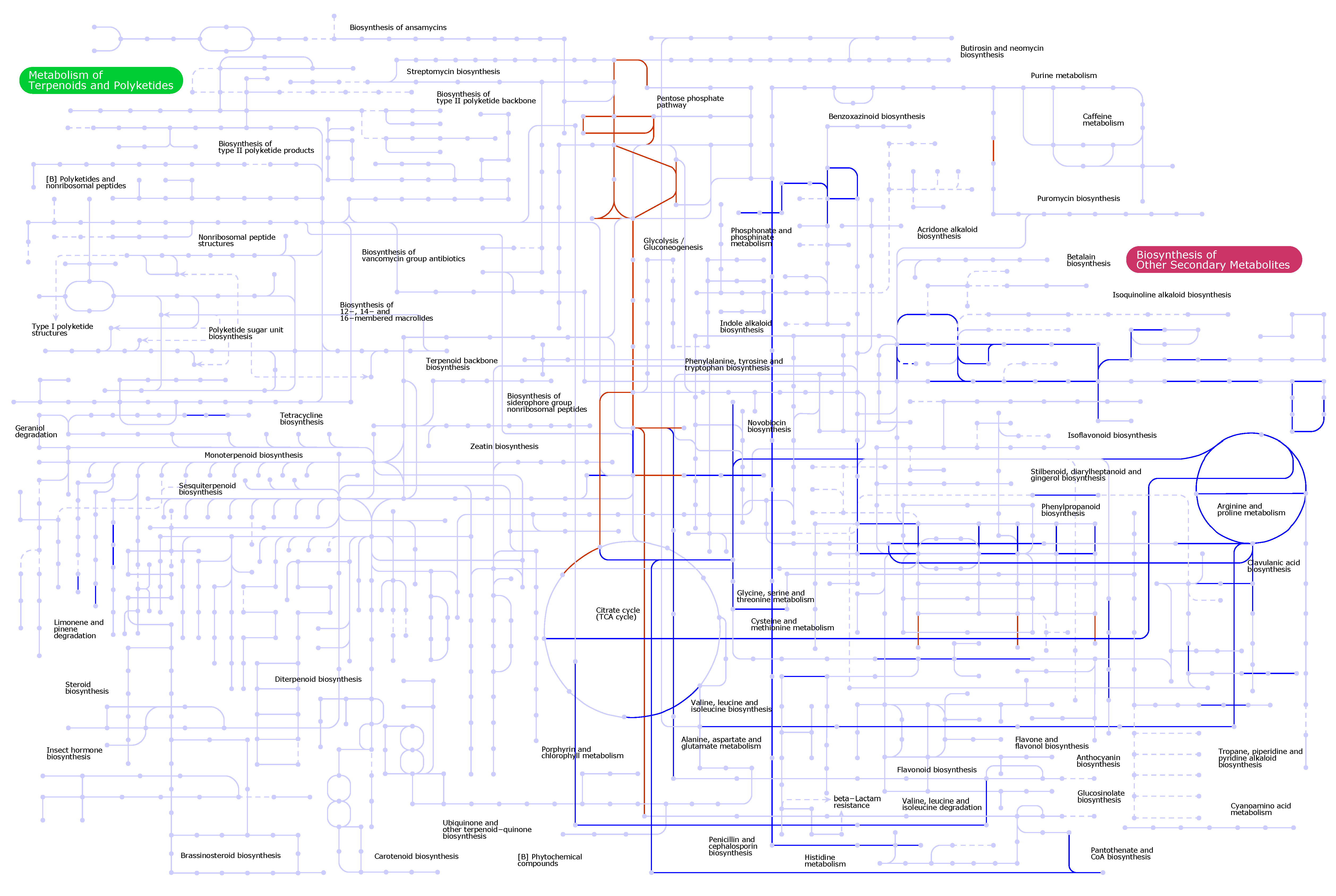

Supplement: Figure S4 — iPath secondary metabolite map showing the different pathways where are involved each evaluated enzyme. Each grey dot represents a metabolite and each colored line represents the different route affected by the enzyme targeted. In red: phosphoglycerate mutase (regulated by eun-MIR396-2). In blue: primary-amine oxidase (regulated by eun-nMIR007). (TIFF) [file pone.0049811.s004.tiff]
