# Supplementary material for: Identification of MicroRNAs from Eugenia uniflora by High-Throughput Sequencing and Bioinformatics Analysis
Source: PLoS One. 2012 Nov 15;7(11):e49811. doi: 10.1371/journal.pone.0049811 (PMC3499529; doi:10.1371/journal.pone.0049811)

**Figure S2.** Predicted secondary structures of the conserved and novel miRNA precursors of *E. uniflora* and the locations of the more abundant mature miRNAs.

eun-MIR156

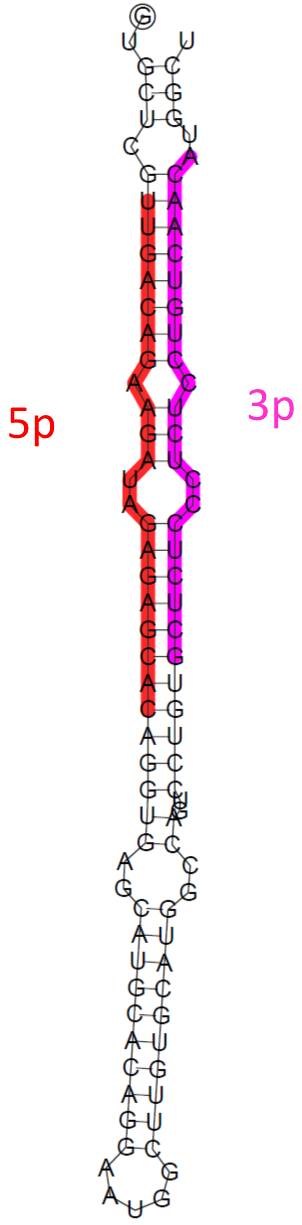


eun-MIR159

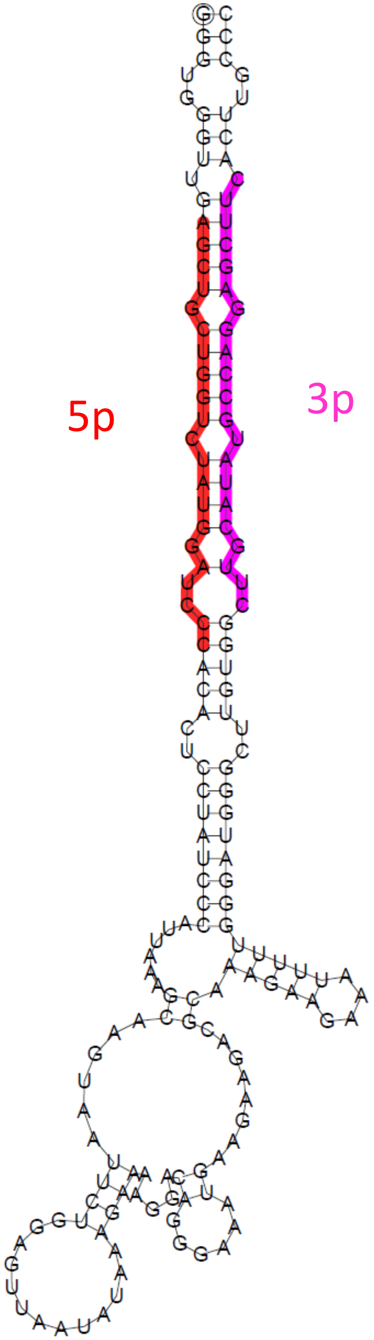

eun-MIR160

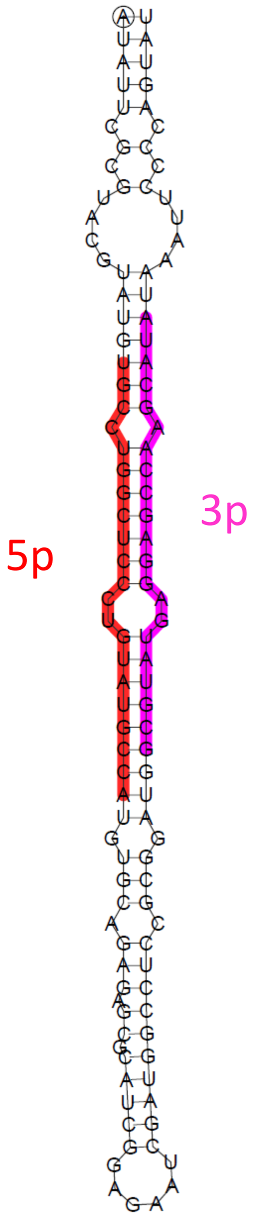


eun-MIR162

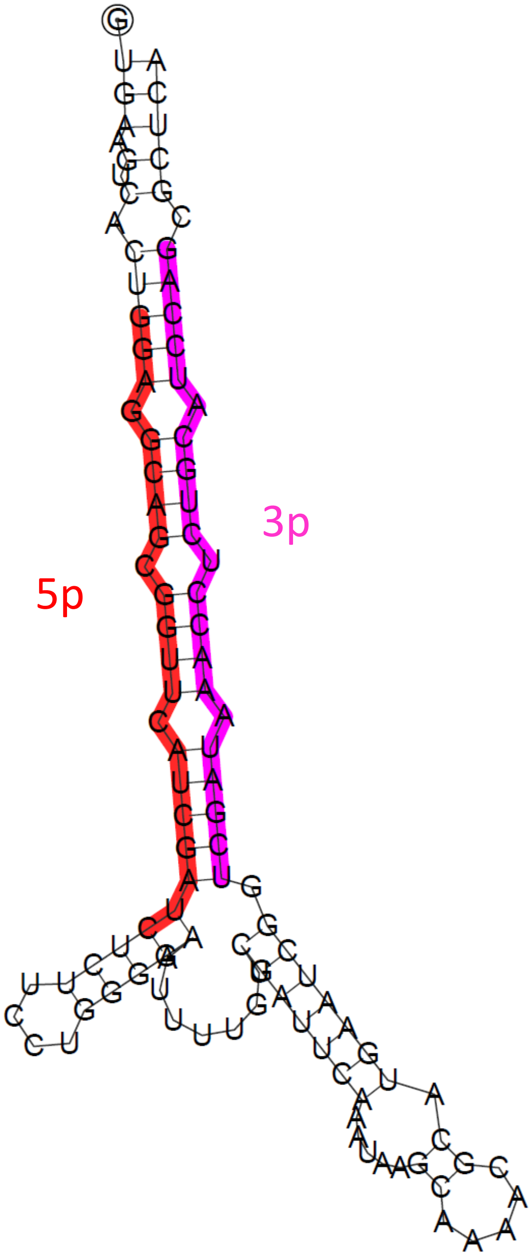

eun-MIR166

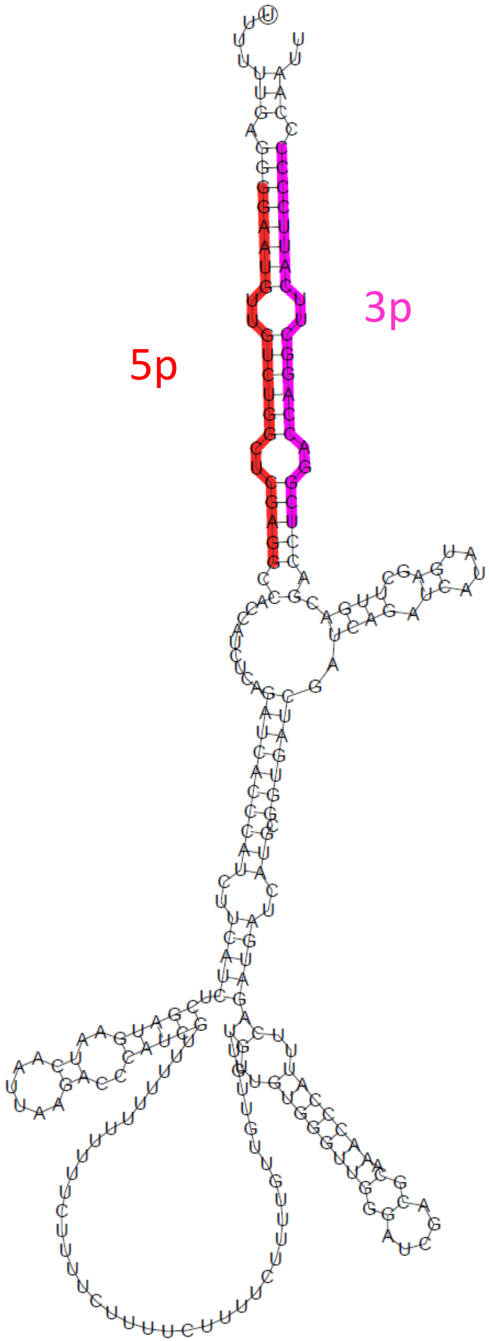


eun-MIR167-1

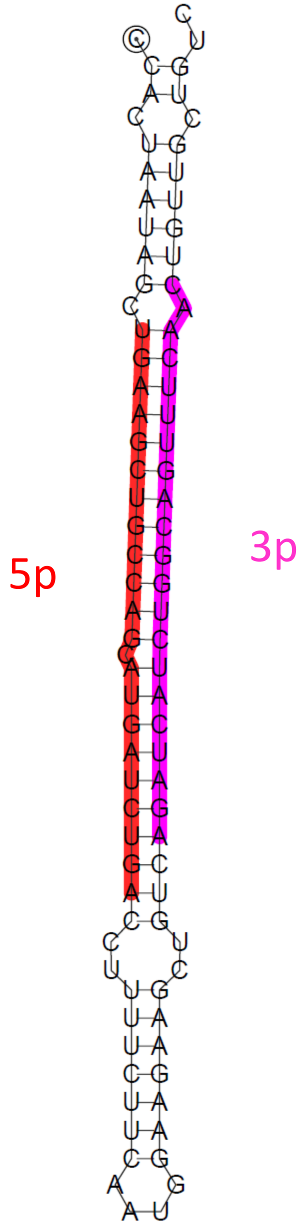

eun-MIR167-2

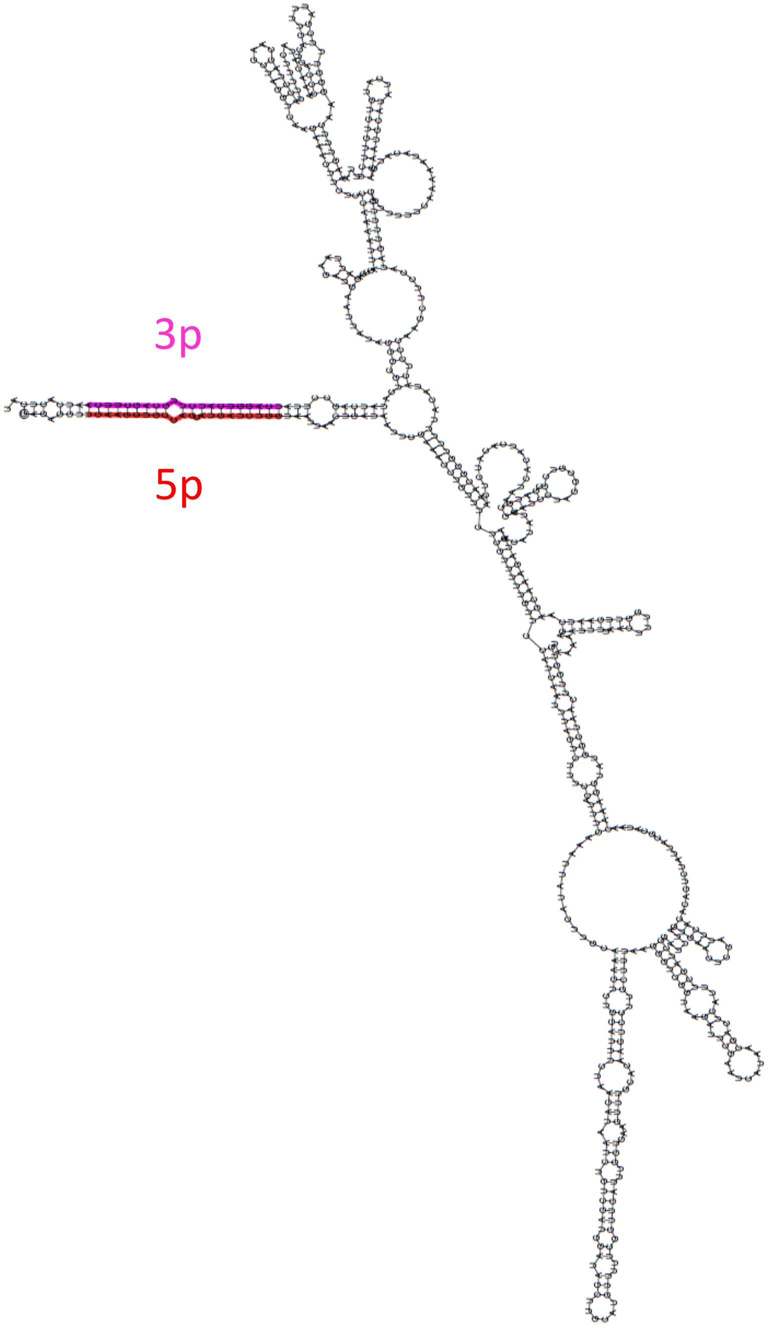


eun-MIR167-3

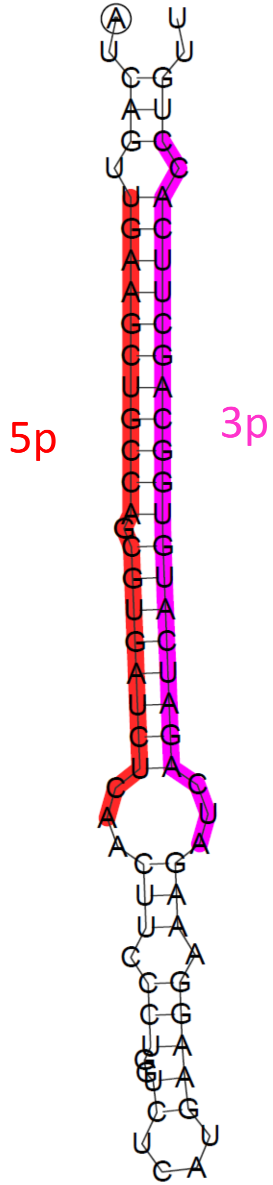

## eun-MIR167-4

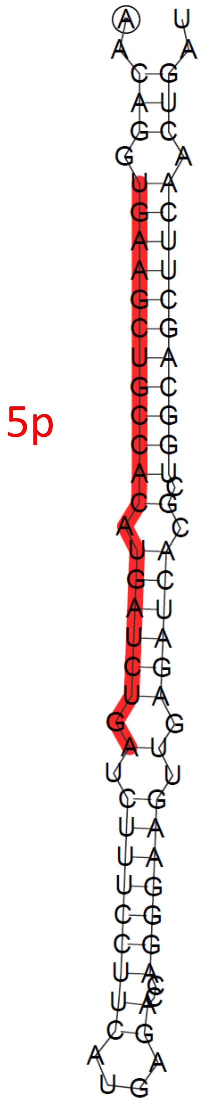


## eun-MIR169

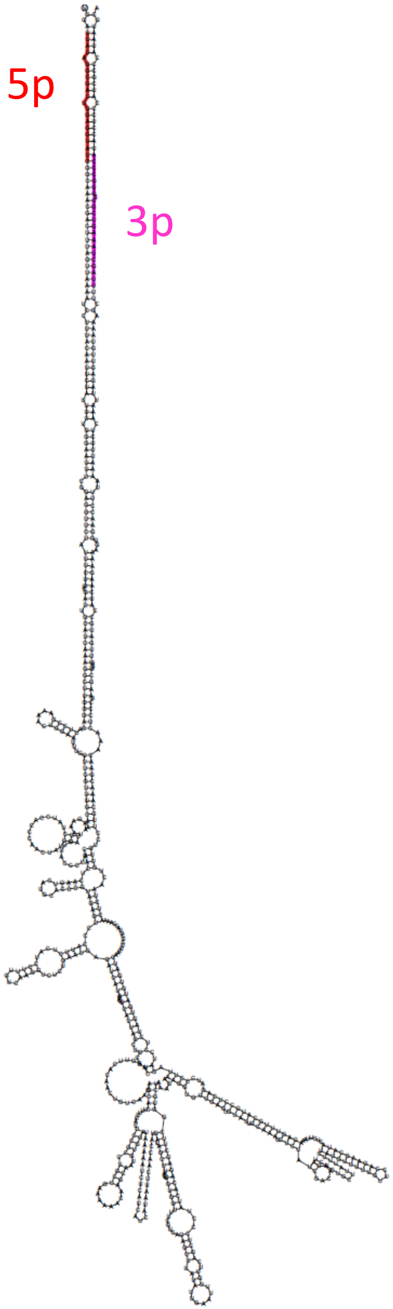

eun-MIR172-1

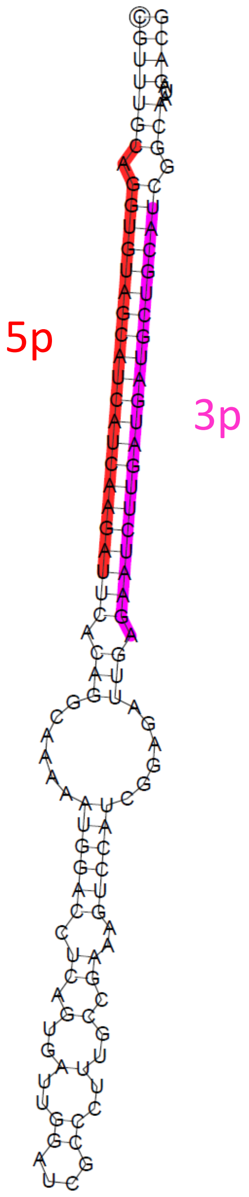


eun-MIR172-1

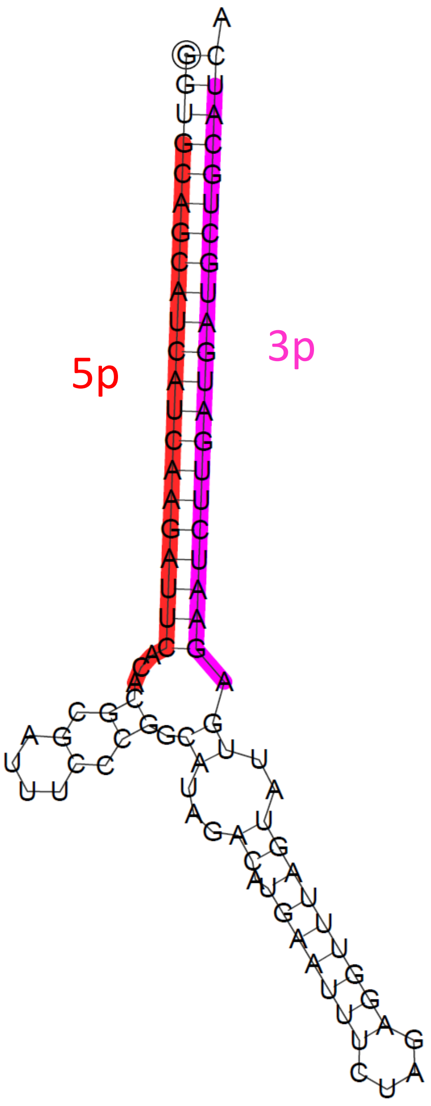

## eun-MIR172-3

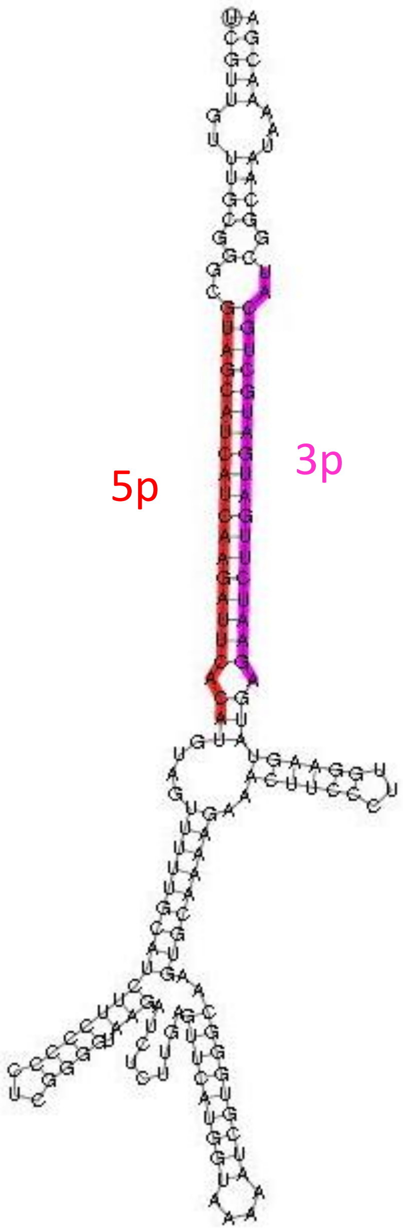


## eun-MIR395

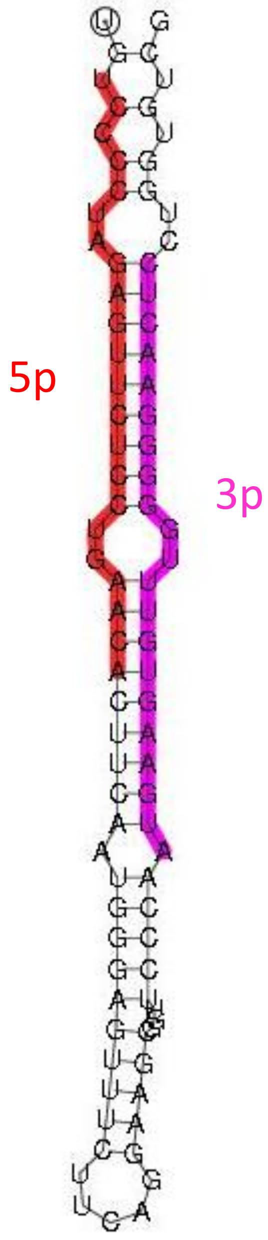

eun-MIR396-1

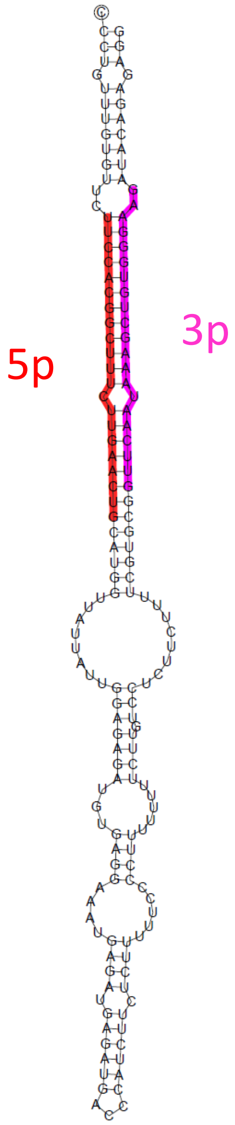


eun-MIR396-2

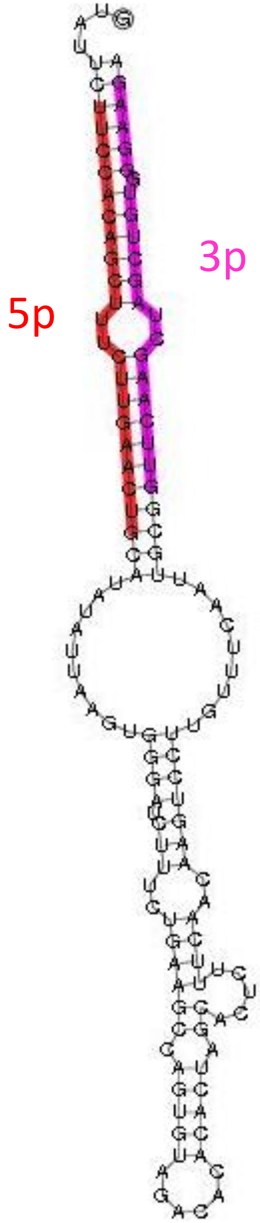

eun-MIR397-1

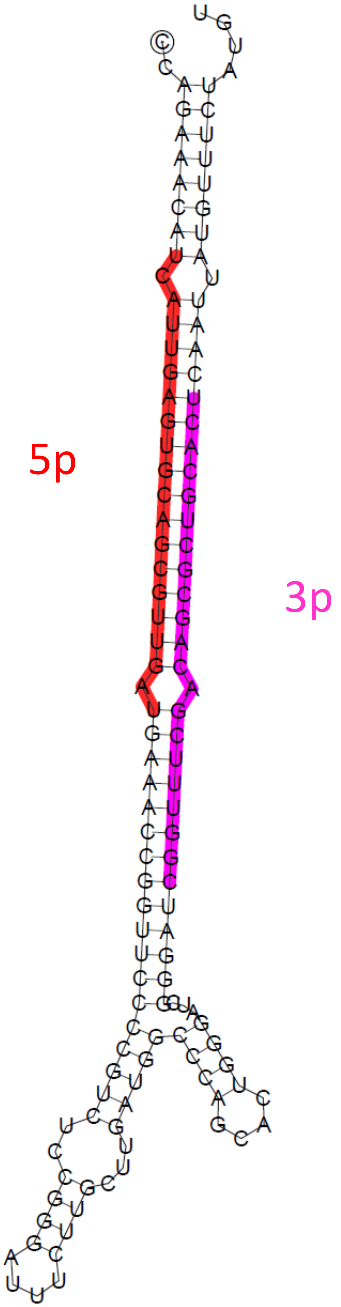


eun-MIR397-2

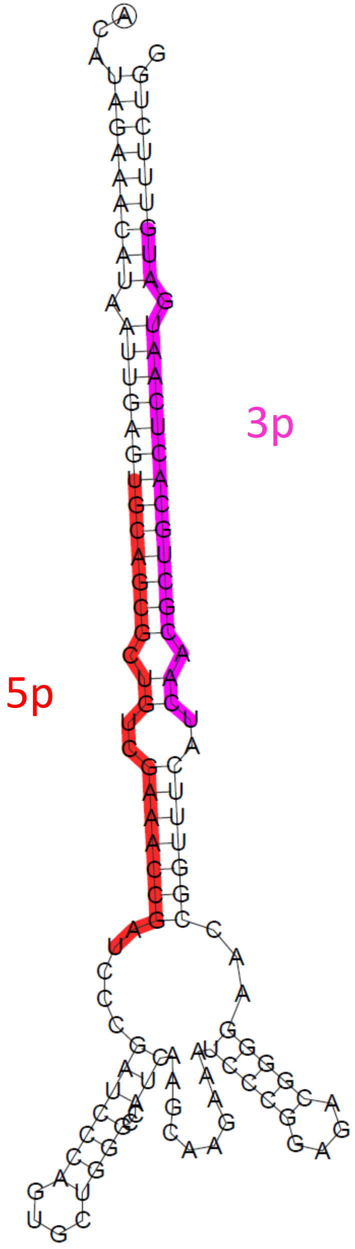

## eun-MIR482-1

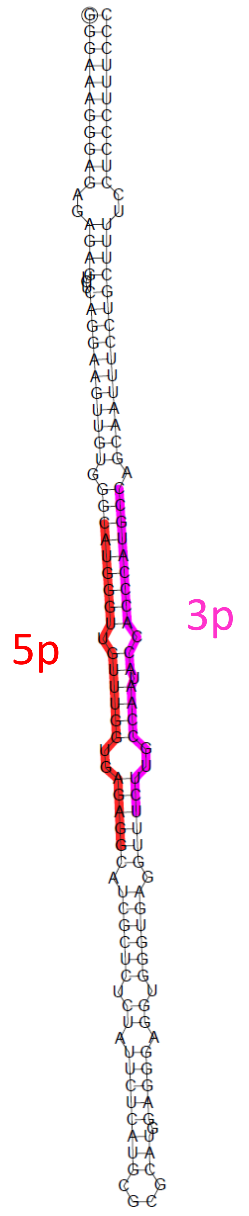


## eun-MIR482-2

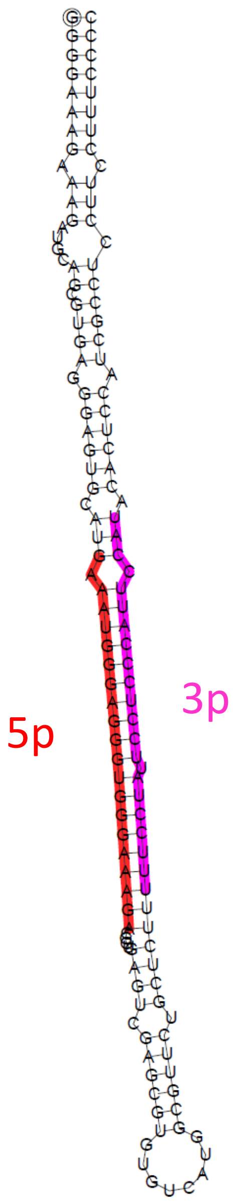

## eun-MIR482-3

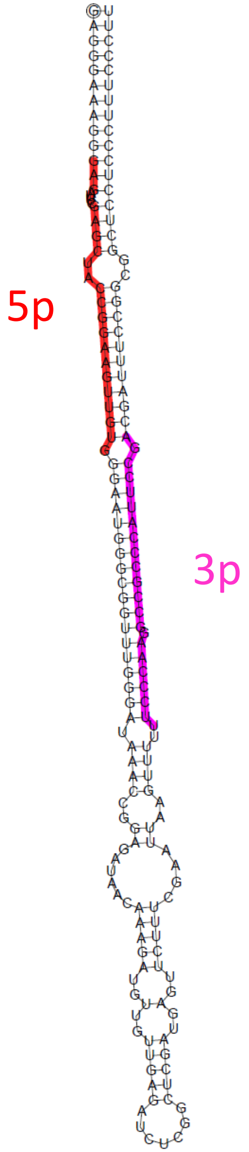


## eun-MIR530

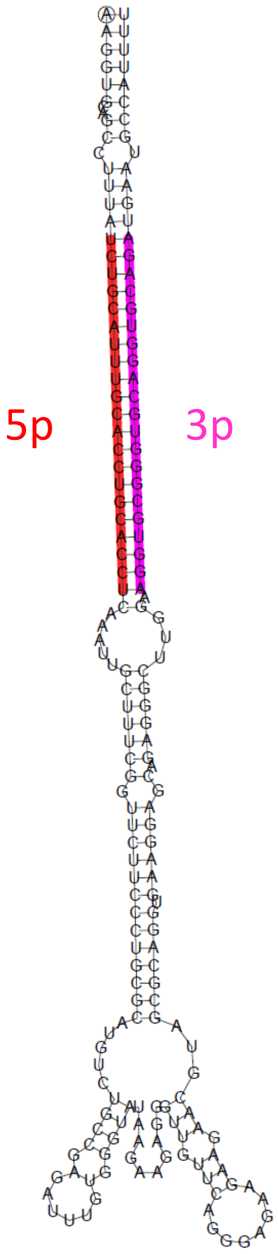

eun-MIR535-1

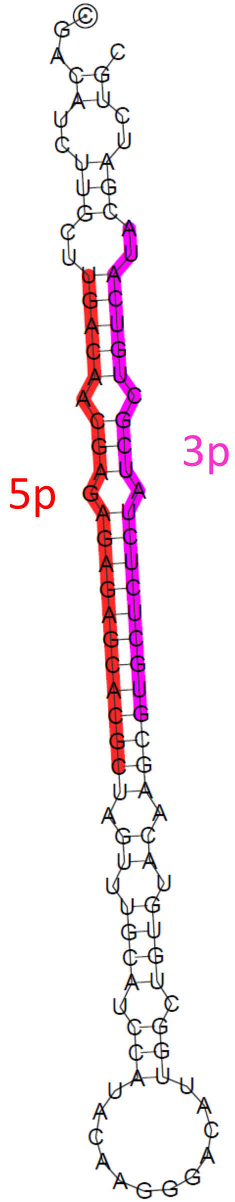


eun-MIR535-2

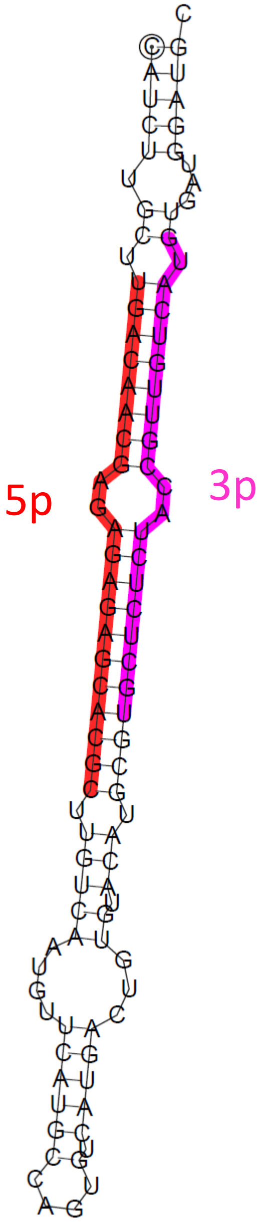

eun-MIR827

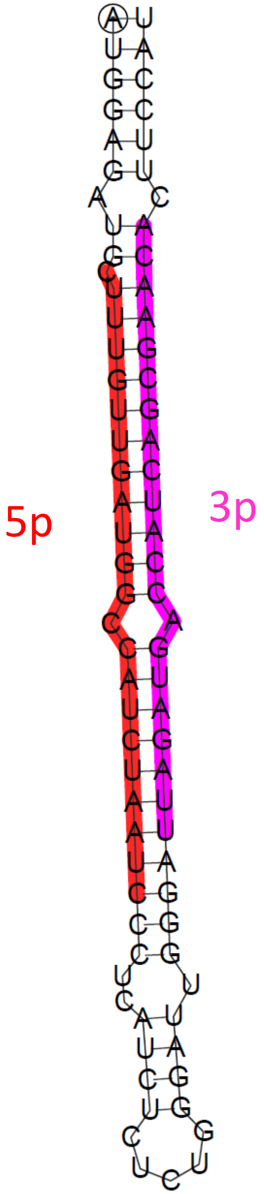


eun-nMIR001-1

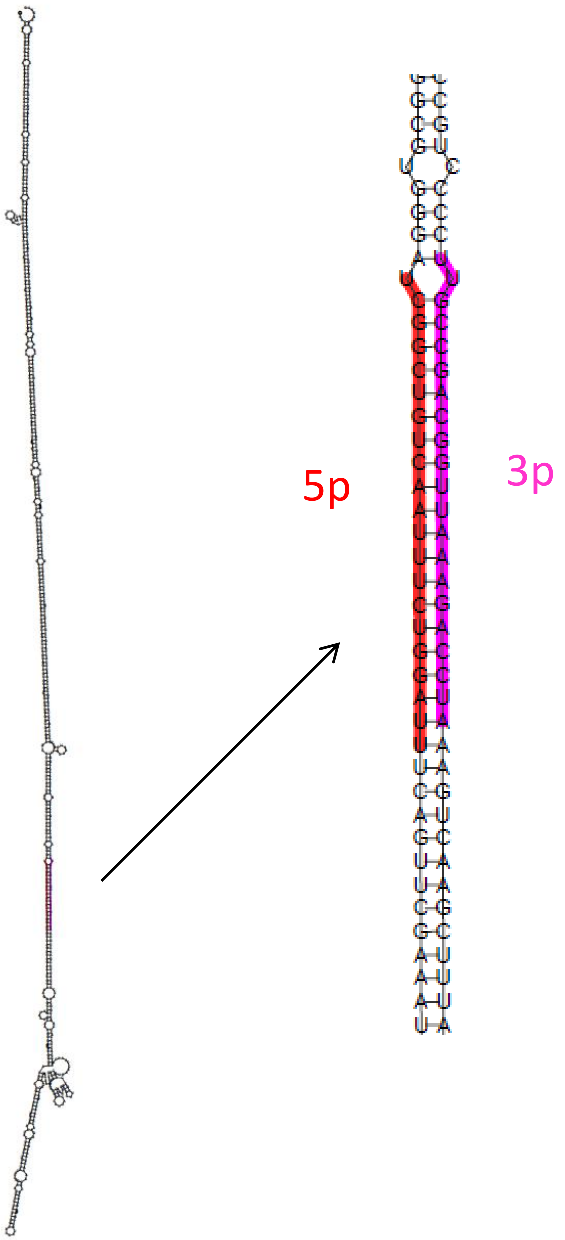

eun-nMIR001-2

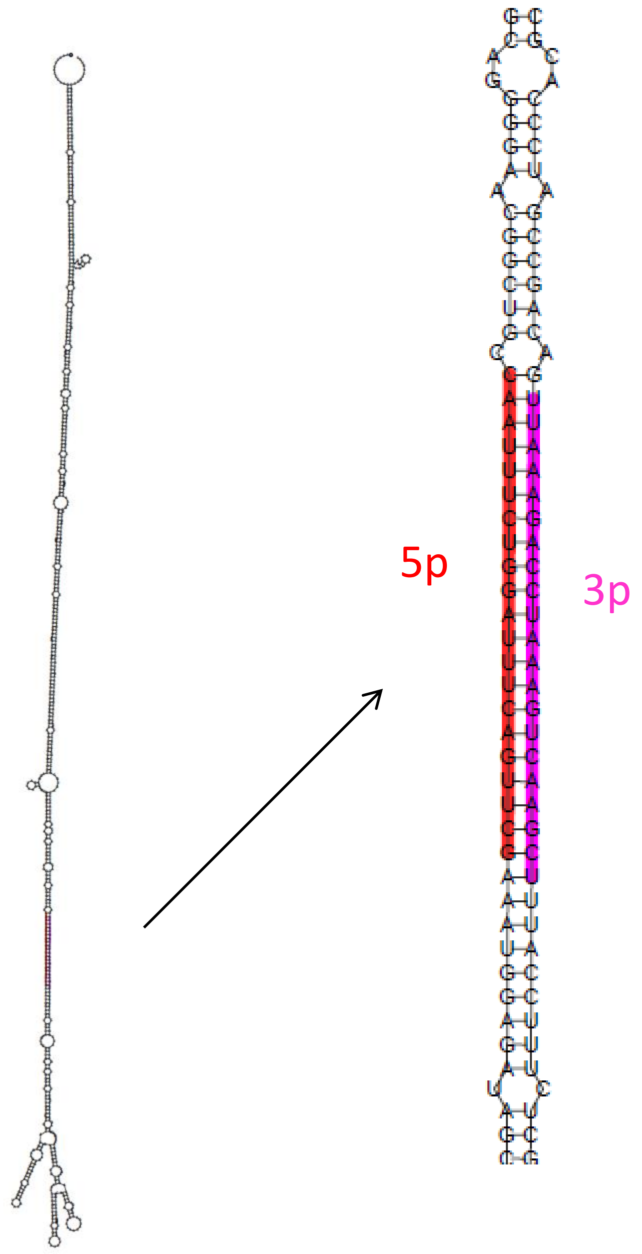


eun-nMIR002

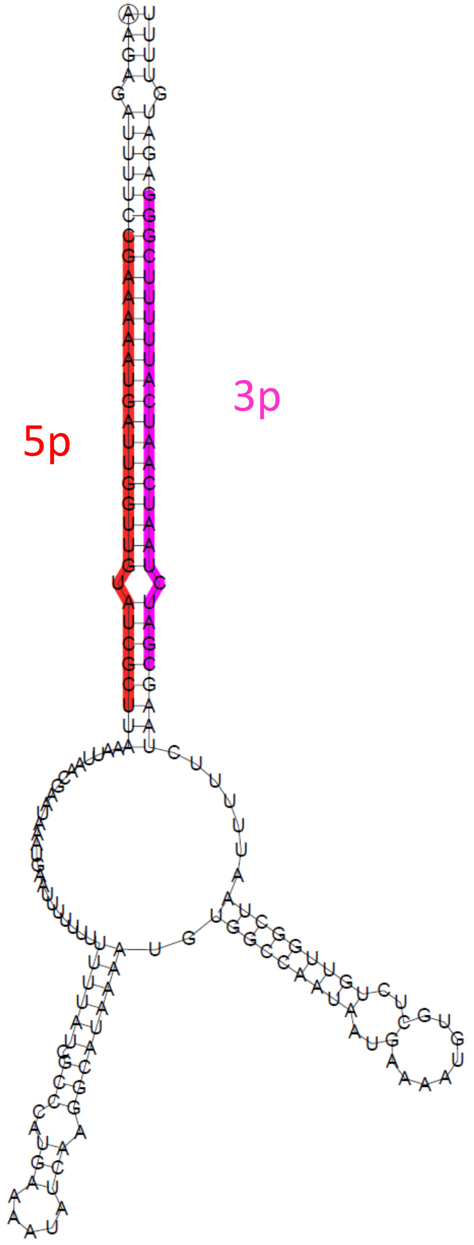

# eun-nMIR003

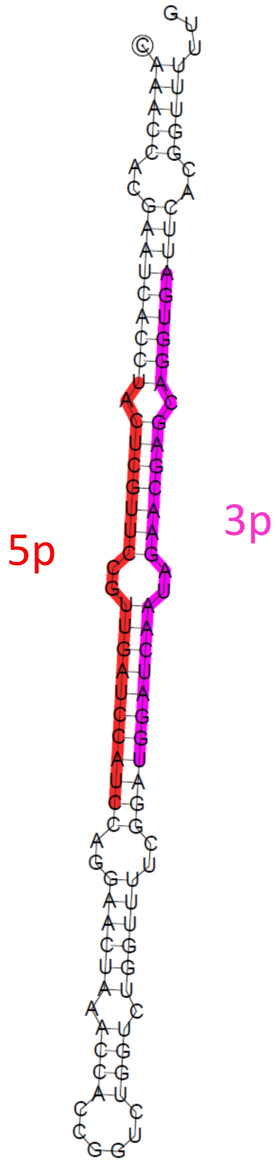


# eun-nMIR004-1

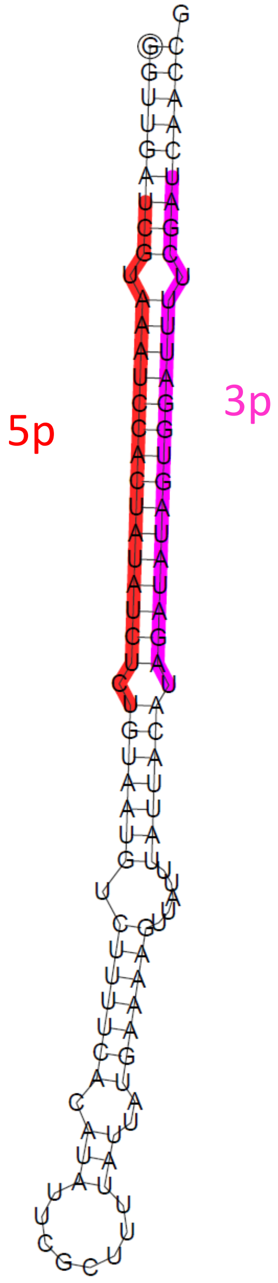

eun-nMIR004-2

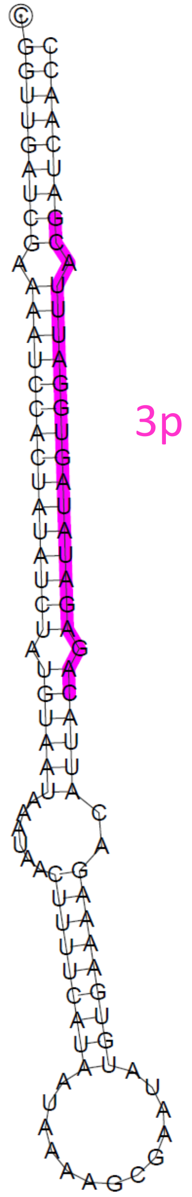


eun-nMIR005

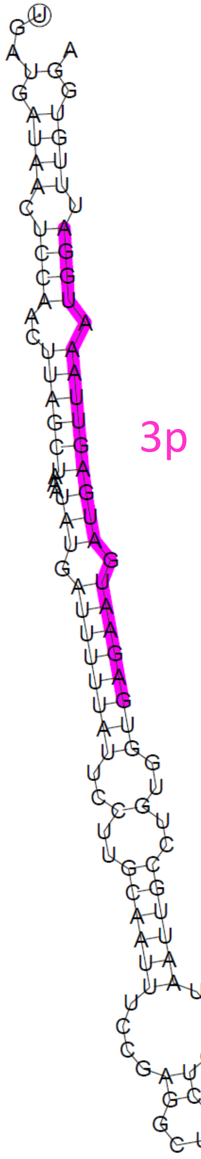

eun-nMIR006

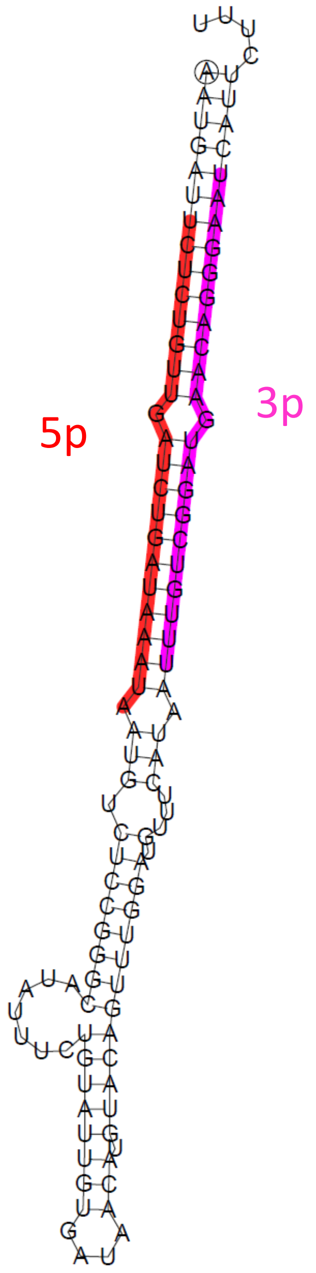


eun-nMIR007

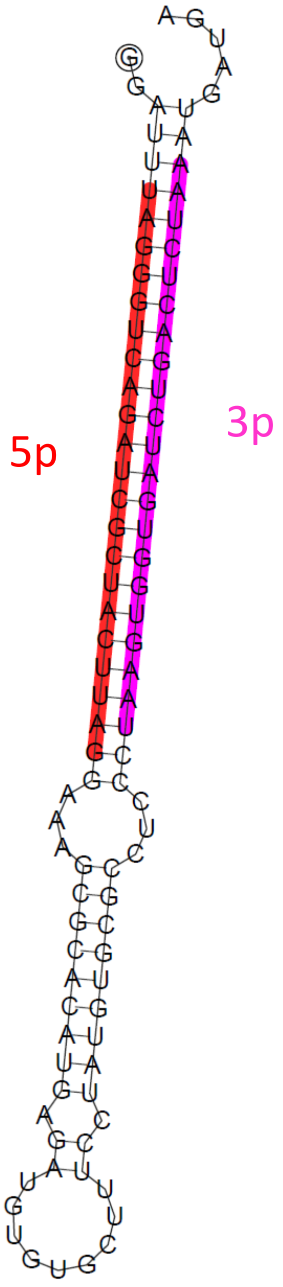

# eun-nMIR008

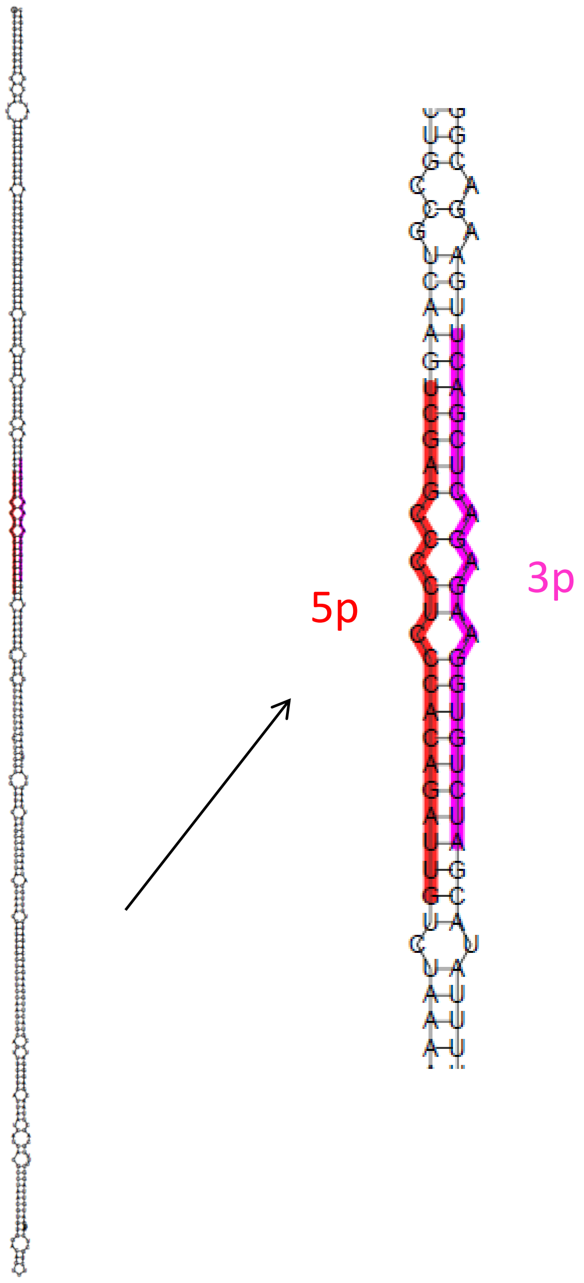


# eun-nMIR009

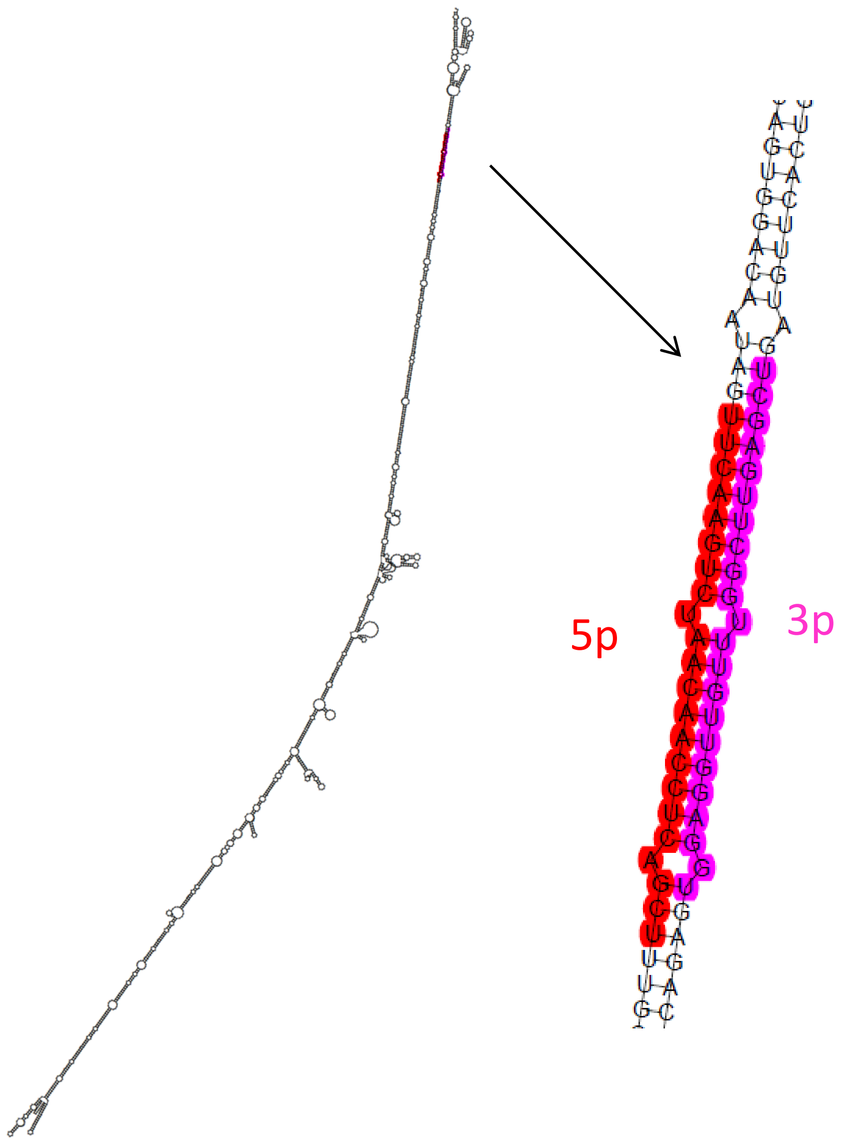

# eun-nMIR010

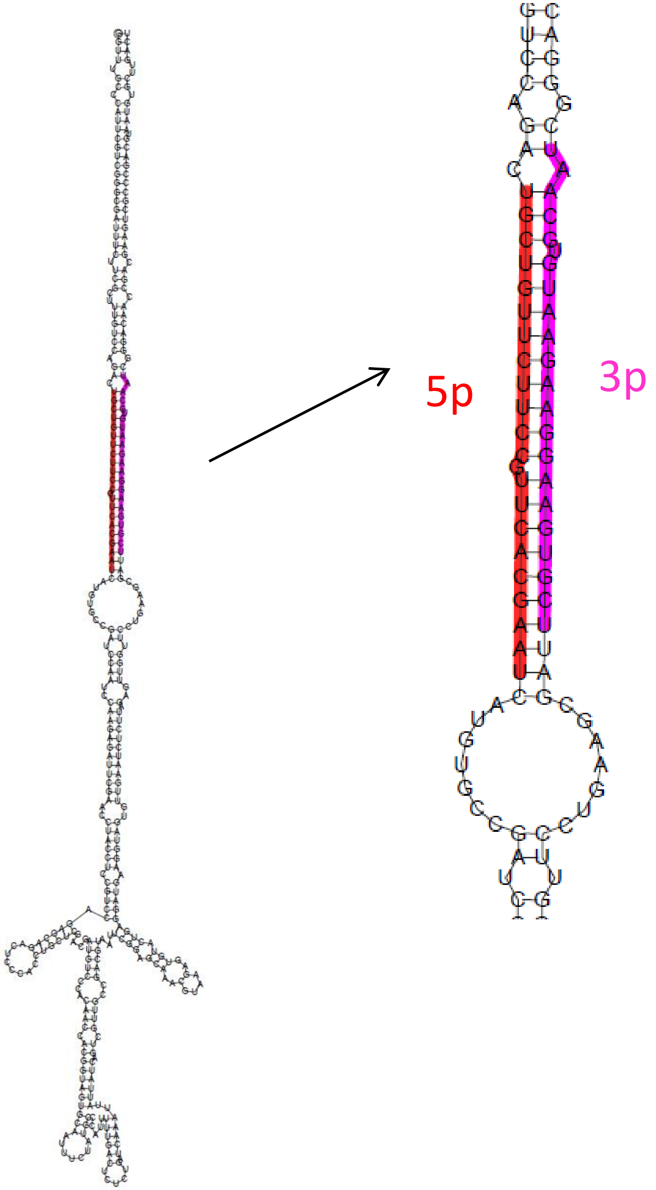


# eun-nMIR011

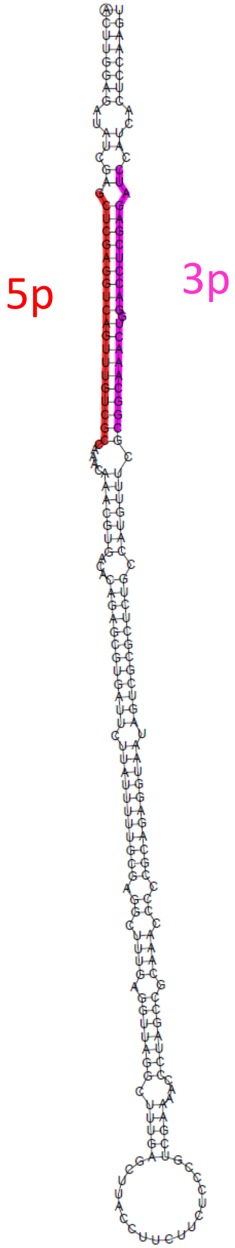

# eun-nMIR012

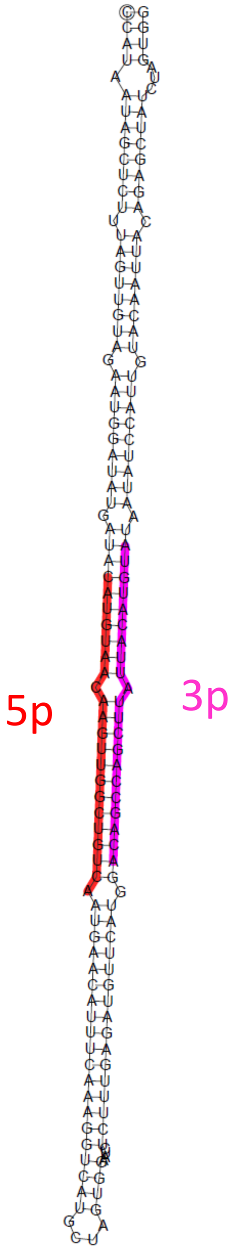


# eun-nMIR013

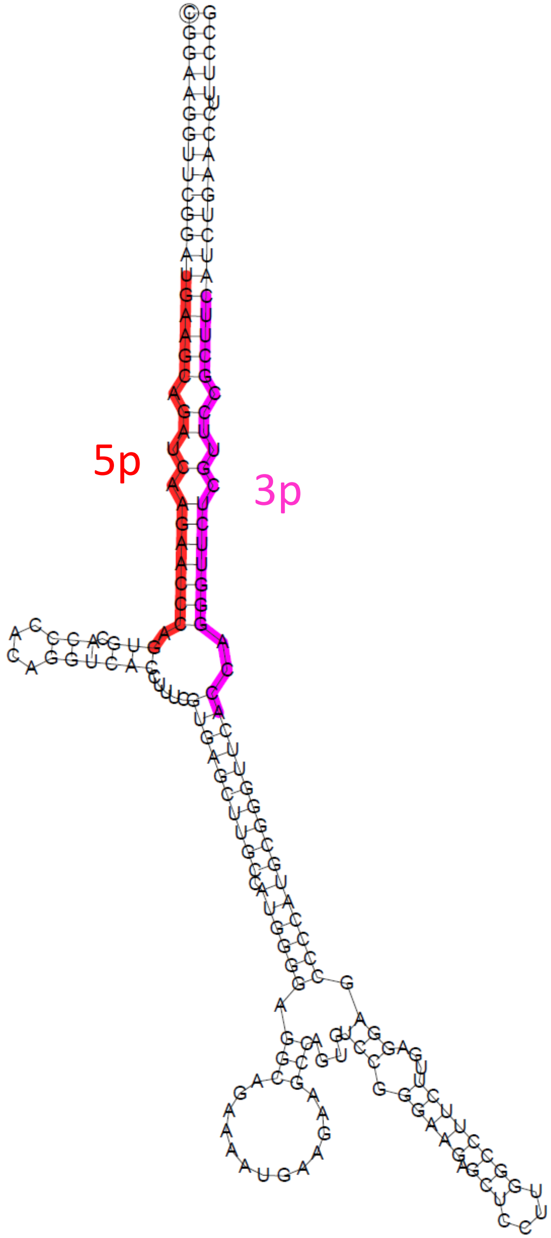

# eun-nMIR014

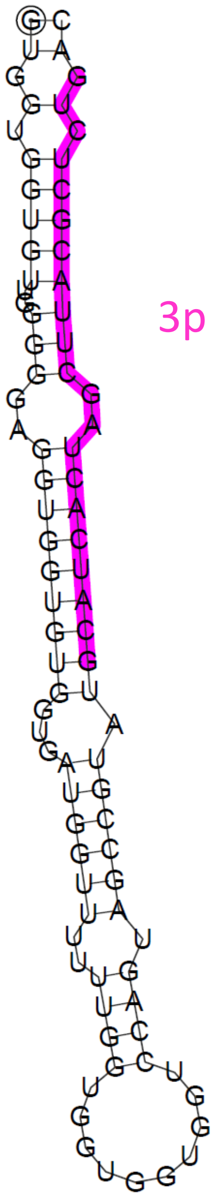


# eun-nMIR015

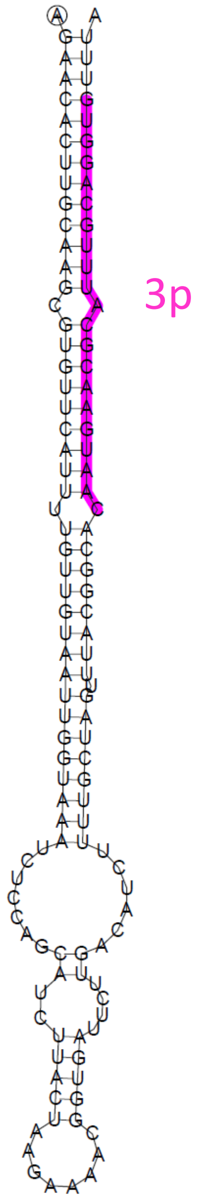

Supplement: Figure S2 — Represetation of the predicted secondary structures of the conserved and novel miRNA precursors of E. uniflora and the locations of the more abundant mature miRNAs. (PDF) [file pone.0049811.s002.pdf]
